# The evolution of word retrieval errors during semantic feature-based therapy in bilingual aphasia

**DOI:** 10.1017/S1366728925100370

**Published:** 2025-08-11

**Authors:** Michael Scimeca, Claudia Peñaloza, Erin Ann Carpenter, Manuel Jose Marte, Marissa Russell-Meill, Swathi Kiran

**Affiliations:** 1 Department of Speech, Language, and Hearing Sciences, Boston University, MA, USA; 2 Department of Cognition, Development and Educational Psychology, Faculty of Psychology, University of Barcelona, Barcelona, Spain; 3 Institute of Neurosciences, University of Barcelona, Barcelona, Spain; 4 Cognition and Brain Plasticity Unit, Bellvitge Biomedical Research Institute-IDIBELL, L’Hospitalet de Llobregat, Barcelona, Spain

**Keywords:** bilingual aphasia, anomia, error analysis, treatment outcomes, cross-language activation

## Abstract

Bilinguals with aphasia routinely experience anomia in one or both of their languages that may be ameliorated by language treatment. Traditionally, treatment response has been captured by binary scoring systems that measure the presence or absence of improvement without examining how word retrieval attempts may change over time as a function of treatment. This study analyzed word retrieval errors and subsequent treatment outcomes for a group of 48 Spanish-English bilinguals with aphasia to determine if longitudinal error patterns could capture language recovery. Results revealed naming improvement for trained words in the treated language and translations of trained words in the untreated language. Specific types of word errors at baseline were associated with overall improvement in both languages; furthermore, patterns of responses changed over time as a function of lexical-semantic treatment. These results demonstrate that error analyses may characterize bilingual treatment outcomes and provide new evidence for mechanisms of impaired word retrieval.

## Highlights

- Semantic feature treatment may improve naming in a treated and untreated language.
- Pretreatment naming errors are associated with overall improvement in therapy.
- Change in naming errors suggests improvement beyond traditional therapy scoring.
- Error analyses reveal complex word retrieval strategies in bilingual aphasia.

## 1. Introduction

Bilingual aphasia is characterized by deficits in one or more languages that differ across individuals according to the site and severity of their injury (Fabbro, 2001; Paradis, 2004). In the United States, bilinguals with aphasia (BWA) also frequently identify as members of diverse minority groups whose racial, ethnic and linguistic backgrounds may influence their pre- and post-injury language usage patterns and preferences (Scimeca et al., 2022). Despite this diversity in impairment profiles, BWA frequently present with anomia in one or more of their languages and therefore produce a variety of speech and language errors during everyday conversations. Word finding problems and other communication deficits can negatively impact an individual’s sense of independence in social situations, often leading to a depressed mood and an overall decreased quality of life. These negative consequences of aphasia may be more pronounced for bilingual individuals who may experience deficits in both languages that can constrain their social participation across different sociolinguistic environments.

Previous studies have established the efficacy of language intervention for BWA delivered in their first (L1) or second (L2) language (for a review, see Ansaldo & Saidi, 2014; Faroqi-Shah et al., 2010; Goral et al., 2023; Kohnert, 2009; Peñaloza & Kiran, 2019). Some of the most robust direct treatment outcomes have been observed following semantic feature-based treatments (SFTs) for anomia. SFTs are a broad class of naming therapies, including semantic feature analysis (Boyle & Coelho, 1995) and similar semantic feature verification treatments (Kiran & Thompson, 2003; Kiran et al., 2009), that involve a series of tasks designed to strengthen the semantic-conceptual network with the aim of improving word retrieval on a set of trained items while promoting generalization to semantically related words. In bilingual aphasia, the delivery of SFTs has been associated with significant improvements on trained words in a treated language as well as cross-language generalization – or improvement on untrained words in an untreated language (Croft et al., 2011; Edmonds & Kiran, 2006; Kiran & Iakupova, 2011; Kiran & Roberts, 2010; Kiran et al., 2013; Peñaloza et al., 2021; Scimeca et al., 2024).

These findings have been further substantiated by a large systematic review and meta-analysis of bilingual treatment studies that identified robust treatment effects in the treated language as well as small, but significant, cross-language generalization effects (Goral et al., 2023). These patterns were even observed for within-language generalization to untrained, semantically related words and cross-language generalization to translation equivalents of semantically related words. In another review focused on bilingual anomia treatment, Lee and Faroqi-Shah (2024) identified various patterns of improvement. Across 17 studies, the authors reported medium effect sizes for directly trained items and marginal effect sizes for within-language generalization to both untrained related and unrelated words. In the untreated language, there was a marginal effect size for cross-language generalization to translations of directly trained items only. Both reviews suggest that naming improvement for directly trained words may spread to other word categories, resulting in various patterns of improvement in one or more languages following treatment for BWA.

In some studies, however, BWA have shown no direct treatment gains following SFT in either the treated or untreated languages (Croft et al., 2011; Kiran et al., 2013; Peñaloza et al., 2021). Others have reported significant naming improvement for a specific set of trained words but no cross-language generalization to their translation equivalents (Croft et al., 2011; Kiran & Roberts, 2010). Finally, patterns of within-language generalization to untrained, semantically related words in the treated language as well as cross-language generalization to translations of semantically related words in the untreated language are reported in the literature (Edmonds & Kiran, 2006; Kiran et al., 2013), although these patterns have not always been observed.

Given the degree of variability in within-language and cross-language generalization effects, it is difficult to predict patterns of treatment outcomes or ascertain whether only specific sets of outcomes are to be expected following bilingual SFT. Understanding how to maximize direct treatment effects and generalization effects may be better informed by studies that employ longitudinal analyses to examine how naming dynamically changes over the course of SFT. For example, Scimeca et al. (2024) demonstrated a robust treatment effect for trained words in the treated language and a weaker but still significant effect of cross-language generalization to translation equivalents in the untreated language for a group of 34 BWA across 16 naming timepoints. The magnitude of these improvements increased as a function of treatment session, with milder participants showing the most robust treatment gains. Braun and Kiran (2022) found similar results for 30 monolinguals with aphasia (MWA) who completed SFT. Their study demonstrated that participants who had more intact semantic and phonological processing skills at baseline improved the most by the end of the intervention. Additionally, more complex words from a lexical-semantic standpoint (e.g., low lexical frequency) and less complex words from a phonological standpoint (e.g., short length) improved the most in treatment.

To answer some of these outstanding clinical questions – such as how to better predict individual responsiveness to treatment and what factors facilitate generalization to untrained items – may require moving beyond traditional scoring practices, where participant responses are only coded as correct/incorrect. These binary accuracy systems measure the presence or absence of a treatment effect, but they obscure how naming responses may change over time, especially if naming performance is assessed throughout the course of treatment. Error analyses of incorrect naming responses that capture changes in word retrieval may illuminate patterns or gradations of naming improvement, which are missed by more traditional binary scales. In addition to measuring treatment improvement, error analyses may reveal the underlying mechanisms of lexical activation and the overall (de)composition of the lexical-semantic network in aphasia (Martin et al., 1996; Schwartz et al., 2006; Schwartz, 2014), as disturbances in lexical activation due to noise or weakened representations within or between levels of linguistic processing can lead to distinct word retrieval errors. To outline how the present study used error analyses to investigate lexical activation and language recovery in bilingual aphasia, the following sections (i) highlight principles of bilingual lexical access and linguistic processing that provide the foundation for assessing error types based on possible loci of impairment and (ii) synthesize findings from previous studies that have implemented error scoring to examine anomia in MWA and BWA.

### 1.1. Word production in bilinguals

In recent decades, various models of lexical access have been proposed to account for activation and retrieval of lexical units in healthy bilinguals (Costa et al., 2006; de Groot, 1992; Dijkstra et al., 2019; Kroll & Stewart, 1994; van Hell & de Groot, 1998). In general, these theoretical frameworks agree that language processing in bilinguals proceeds via a shared lexical-semantic network in which language activation spreads in a language nonselective manner. Here, we focus on bilingual speech production models that have wide implications for accounting for and predicting word retrieval errors in aphasia.

Although most models of speech production assume three levels of representation, they differ in their hypotheses about the degree and extent to which spreading activation drives word retrieval (Costa et al., 2006). For example, discrete models and their bilingual equivalents have argued that activation spreads from the lexical level to the phonological level only for the word that is ultimately selected for production (Levelt et al., 1991, 1999). On the other hand, cascading models of activation posit that activation of any nodes in the lexical level will propagate to applicable phonological units in the sublexical level (Caramazza, 1997; Dell, 1986; Martin et al., 1996).

Regarding language selection, increasing evidence suggests that when a speaker plans to produce words in a given language, the other, nontarget, language is activated in parallel (Colomé, 2001; Costa et al., 1999; Costa, 2005; de Bot, 1992; La Heij, 2005). Control processes are needed to constrain this parallel activation and suppress the nontarget language in context (Abutalebi & Green, 2007; Costa & Santesteban, 2004a; Costa & Santesteban, 2004b; Green, 1998; Kroll et al., 2008) especially if we consider that (i) lexical activation of words in the nontarget language may flow to phonological units supporting these translation equivalents (Costa et al., 2000) and (ii) bidirectional activation (i.e., feed-backward) from phonological units to corresponding lexical nodes is possible in both languages. With these additional stipulations, not only would “dog” and its Spanish translation *perro* be activated, but subsequent activation of the phonological units for these words would propagate back to the lexical level and activate phonologically related words in both languages as well.

### 1.2. Error analyses in aphasia treatment

The motivation for error analyses in aphasia stems from Dell’s interactive activation model of lexical processing (Dell, 1986; Dell et al., 1997), which posits that word retrieval errors occur due to damage to various levels of processing in a hierarchical network. Damage in this network can be localized to semantic, lexical and/or phonological levels of processing or the connections between them based on the frequency and nature of word retrieval errors. For example, unrelated errors suggest damage to the connections between semantics and lexemes, while lesions that disrupt activation throughout the system could result in mixed semantically- and phonologically-related errors.

This framework serves as the theoretical foundation for anomia treatment in aphasia, which focuses on strengthening spreading activation within the lexical-semantic network. As a result, changes in the nature and frequency of naming errors may serve as a measure of progress in therapy and provide insights into the mechanisms underlying treatment-induced recovery in aphasia. To date, most studies examining changes in error patterns across treatment phases (e.g., pre- versus post-treatment) have yielded mixed results among MWA (Abel et al., 2007; Kendall et al., 2013; Minkina et al., 2016) and BWA (Edmonds & Kiran, 2006; Keane & Kiran, 2015; Kurland & Falcon, 2011; Li et al., 2020). For instance, while omission errors tend to decrease posttreatment (Abel et al., 2007; Kendall et al., 2013; Minkina et al., 2016), suggesting general improvements in lexical access abilities, other studies have reported increases in omission errors (e.g., Edmonds & Kiran, 2006) and neologisms (Li et al., 2020). Variable changes to the semantic level have also been documented, as some individuals have produced fewer neologisms and semantic errors over time, while others have displayed greater semantic errors posttreatment (Edmonds & Kiran, 2006). For bilingual aphasia in particular, error analyses provide a unique opportunity to explore the interactions between linguistic representations in L1 and L2 and investigate how treatment in one language influences performance in the untreated language, potentially elucidating the mechanisms underlying cross-language generalization in this population. Prior work in this area has provided evidence for both increases (Edmonds & Kiran, 2006; Keane & Kiran, 2015; Kurland & Falcon, 2011) and decreases (Li et al., 2020) in nontarget language errors following treatment, although such errors are not always observed in BWA (Li et al., 2020).

Several aspects of these studies have made it difficult to draw strong conclusions about the relationships between the evolution of errors and overall treatment response. First, relatively small sample sizes among existing studies have contributed to variable patterns of error production, which is undoubtedly influenced by language background (i.e., monolingual versus bilingual) and individual differences in aphasia profiles. Second, studies have employed various treatment paradigms that differ not only in the type of intervention but also in the number of phases and frequency of delivery – factors that could influence the types of errors produced over the course of treatment. Finally, previous studies have only analyzed differences in total errors between treatment phases rather than examining how errors changed within treatment phases.

### 1.3. The current study

To address these gaps in the literature, the present study examined naming errors from 48 Spanish-English BWA who completed 20 sessions of SFT. The data were collected and scored at the item level across sessions – using a bilingual scoring framework developed by Kiran et al. (2014), which will be reviewed below – to provide the largest and most granular analysis of word retrieval errors for individuals with aphasia to date.

**Figure 1.**
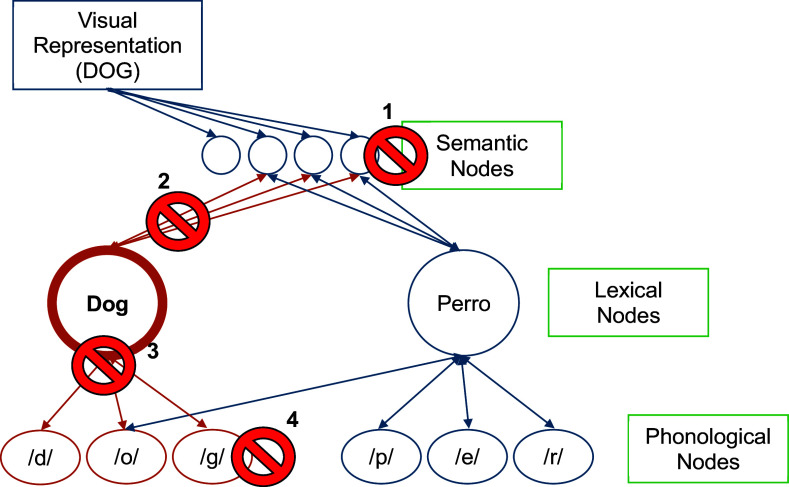
A visual representation of cascading activation when retrieving the word “dog” across three levels of representation in English and Spanish is presented. Hypothesized loci of damage represented by numbers 1–4 are included to contextualize potential types of error responses. Damage at 1 suggests little to no semantic access in the language system. Level 2 represents incomplete semantic access. Level 3 demonstrates incomplete lexical access, noisy phonological activation or a combination of the two. Level 4 suggests typical or completed lexical-semantic access but incomplete phonological access.

Kiran et al. (2014) suggested that different types of bilingual naming errors could signal a disruption at different levels of lexical-semantic access. Consistent with Kiran et al.’s (2014) error scoring framework, Figure 1 shows an example of the word retrieval process for “dog” in English that can be used to visualize possible errors given hypothesized loci of damage to the bilingual lexical-semantic system. At the first level of damage, underspecified semantic representations (Lambon Ralph et al., 2002) would likely cause word retrieval errors that lack substantial contextual information such as omissions (i.e., no responses) or errors with no discernible connection to the visual target such as neologisms or perseverations (level 1). Incomplete semantic access – due to damage to the connections between semantic and lexical nodes (level 2) – would likely result in unrelated, circumlocution and semantic errors, which vary from least (unrelated) to most (semantic) overlap with the intended target word. Damage to the lexical nodes or the connections between lexical nodes and corresponding phonological nodes could result in mixed errors that contain evidence of incomplete semantic access, incomplete phonological activation or a combination of the two (level 3). Finally, phonemic errors, motor speech errors and accent-influenced responses reflect successful semantic access but varying degrees of dysfunction or cross-language interference at the phonological access level (level 4).

A key characteristic of the cascading activation in Figure 1, and thus word retrieval errors, is that responses can occur in either language. For example, while retrieving the word “dog”, a bilingual individual with aphasia could produce a semantic error, “wolf” in English or its Spanish translation, *lobo.* Kiran et al.’s (2014) coding system captures the richness of these responses by combining the category of the response (e.g., semantic error) and the language in a single score. Using this framework, they assessed poststroke language performance and demonstrated that Spanish-English BWA produced primarily semantic and circumlocution errors in the target and nontarget language. These results were consistent with general patterns of lexical-semantic impairment in aphasia and suggested that the most prominent error types were consistent with semantic access impairments.

### 1.4. Research aims

Using the framework from Kiran et al. (2014), we aimed to investigate whether a comprehensive, group-level analysis of lexical retrieval errors, recorded over the course of bilingual SFT, could illuminate naming improvements beyond a binary scoring system. Our research questions and analyses extend the findings of Scimeca et al. (2024), where we examined treatment outcomes in the treated and untreated language for a subset of 34 BWA included in this study. We sought to address the following research questions:

1. What are the patterns of naming improvement in the treated and untreated language following SFT? Extending our analyses from Scimeca et al. (2024) to include a larger sample, we predicted various rates of improvement for six word sets: trained, semantically related and control words in the treated language and their corresponding translations in the untreated language. For trained items in the treated language, we predicted robust improvement and weaker cross-language generalization to untrained translations. Given spreading activation principles in SFT, we acknowledged that different patterns of within- and cross-language improvement could be observed for semantically related and control words in both languages. For instance, Kiran et al. (2013) reported improvement for semantically related words in both languages after SFT. However, Lee and Faroqi-Shah (2024) only found within-language generalization to semantically related and unrelated words with no evidence of cross-language generalization after various anomia interventions. Overall, we expected trained words and their translations to show more improvement than semantically related items in both languages. Finally, because control words in our study had minimal semantic overlap with trained and semantically related words in each language, we expected little to no improvement in either language.
2. To what extent is naming improvement associated with naming errors at baseline? Kiran et al. (2014) argued that naming errors could be used to understand the severity of damage to various levels of processing. Under their error scoring framework, the most severe errors emerged from damage to the semantic representations themselves because underspecified or noisy representations would disrupt cascading activation at all subsequent levels of processing. Therefore, we predicted that better naming improvement across the six word sets in Question 1 would be associated with error profiles at baseline. More specifically, we expected higher proportions of errors that indicate some level of semantic access (e.g., semantic errors) and lower proportions of errors that indicate little to no semantic access (e.g., omissions) to be associated with better naming improvement. These patterns would suggest that more intact lexical-semantic processing and less severe anomia at baseline are associated with better treatment response (Braun & Kiran, 2022; Scimeca et al., 2024).
3. To what extent do error responses change over time in the treated and untreated language? Broadly, we expected errors reflective of more severe anomia (e.g., no responses, neologisms) to decrease and more semantically- and phonologically-mediated errors to increase over time (e.g., mixed errors) (Kendall et al., 2013) in both the treated and untreated language due to language nonselective spreading activation within the bilingual lexical-semantic system (Costa et al., 2006). Additionally, in the untreated language, we expected an increase in responses that were correct but were produced in the nontarget language (e.g., participant responded *perro* during an English naming probe for “dog”), consistent with predictions from bilingual processing models (Costa et al., 2000) and the capacity for cross-linguistic errors to change over the course of SFT (Edmonds & Kiran, 2006; Kurland & Falcon, 2011; Li et al., 2020).

## 2. Methods

### 2.1. Study design

Data for this study were collected as part of a larger randomized controlled trial (RCT) (Peñaloza et al., 2020) (registered at www.ClinicalTrials.gov, identifier: NCT02916524) investigating the efficacy of a computational model (Peñaloza et al., 2019) in predicting naming treatment outcomes for 48 Spanish-English BWA. As part of the RCT, participants were randomly assigned to receive 20 sessions of SFT in either Spanish or English. Details about group assignment – which are not relevant for the current study – are provided in Peñaloza et al. (2020). The present study used quantitative methods to examine errors during naming probes that were collected longitudinally from RCT participants in both languages using a multiple baseline, within-group design. The present sample of 48 BWA – each contributing naming data across 16 timepoints with 90 items per timepoint (as detailed below) – provided a longitudinal dataset appropriate for the generalized linear mixed-effects models proposed in research questions 1–3. Based on effect sizes observed in our prior work (e.g., Scimeca et al., 2024) and simulation-based benchmarks for estimating power in generalized mixed-effects models (Kumle et al., 2021), we concluded the current study was sufficiently powered to detect treatment-related changes in naming accuracy and error patterns.

### 2.2. Participants

Forty-eight Spanish-English bilinguals with chronic poststroke aphasia (*n* = 1 tumor etiology) contributed data to this study. All participants resided in the U.S. (*n* = 46), Canada (*n* = 1) or Mexico (*n* = 1) during the study and completed all procedures online via Zoom or in person at our laboratory in Boston. All demographic data including information about bilingual language acquisition and proficiency is available in Table 1 and Supplementary Table S1. All participants received SFT for word retrieval deficits through the RCT; treatment outcomes for 34 of these participants were reported previously (Scimeca et al., 2024). Inclusion criteria for the current study were consistent with the RCT such that participants: (i) had a diagnosis of aphasia secondary to an acquired brain injury (ABI) as determined by a neurologist; (ii) were at least six months post-ABI; (iii) were between the ages of 18–85 and (iv) reported at least some degree of proficiency in both Spanish and English prior to aphasia onset. Participants were excluded if they presented with comorbid psychiatric or neurological conditions (e.g., schizophrenia). All demonstrated adequate vision and hearing necessary for completing study procedures. A trained clinician evaluated each participant’s eligibility for inclusion in the study before obtaining written informed consent in accordance with the Boston University Charles River Institutional Review Board (reference number: 4492E).

**Table 1.** Demographic and bilingual language characteristics for Spanish-English BWA

*N* = 48							L1				L2			
							Language use		Language ability rating %		Language use		Language ability rating %	
	Sex	Age	MPO	Edu	L1	L2 AoA	Pre	Post	Pre	Post	Pre	Post	Pre	Post
M	F = 19 M = 29	53.90	48.18	13.97	S = 40 E = 8	13.20	0.53	0.57	0.95	0.59	0.47	0.43	0.83	0.49
SD		15.90	82.38	3.38		11.08	0.28	0.32	0.09	0.21	0.28	0.33	0.20	0.20

*Note.* Values are provided in Means (M) and Standard Deviations (SD). *MPO* = Months post-onset; *Edu* = Education in years; *L1* = first acquired language; *L2* = second acquired language; *AoA* = age of second language acquisition; *S* = Spanish, *E* = English; *Language Use* = proportion of time spent using each language in a typical week; *Language Ability Rating* = self-reported percentage score of language skills in each language where closer to 1 means stronger skills; *Pre* = prestroke; *Post* = poststroke.

### 2.3. Assessment

Participants completed a variety of assessment measures before and after treatment as part of the RCT to characterize their pre- and post-stroke language abilities in English and Spanish. All testing was administered by Spanish-English bilingual clinicians or research assistants. To assess prestroke language proficiency, we administered the Language Use Questionnaire (LUQ; Kastenbaum et al., 2019) to all participants during pretreatment testing. At both testing timepoints, participants also completed the Western Aphasia Battery-Revised (WAB-R; Kertesz, 2007) and its Spanish adaptation (Kertesz et al., 1990) to characterize aphasia severity. Word retrieval was evaluated using the Boston Naming Test (BNT; Kaplan et al., 2001) and its Spanish translation (Kohnert et al., 1998). Finally, semantic processing was assessed using the three-picture version of the Pyramids and Palm Trees (PAPT; Howard & Patterson, 1992). Group-level, pre- and post-treatment scores for the WAB-R, BNT and PAPT are presented in Table 2; individual scores are presented in Supplementary Table S2.

**Table 2.** Clinical assessment scores and treated language information for Spanish-English BWA

*N* = 48				Treated language				Untreated language			
		PAPT (%)		AQ		BNT (%)		AQ		BNT (%)	
	Tx lang	Pre	Post	Pre	Post	Pre	Post	Pre	Post	Pre	Post
*M*	S = 28 E = 20	0.83	0.85	59.41	62.76	0.35	0.39	50.23	53.3	0.25	0.26
SD		(0.14)	(0.15)	(29.39)	(29.74)	(0.28)	(0.27)	(28.51)	(29.96)	(0.27)	(0.28)

*Note.* Values are provided in Means (M) and Standard Deviation (SD). *PAPT =* Pyramids and Palm Trees Percentage Score (/52); *AQ =* Aphasia Quotient, a measure of aphasia severity from the Western Aphasia Battery-Revised (/100); *BNT =* Boston Naming Test Percentage Score (/60); *Tx Lang* = Treatment Language; *S* = Spanish; *E* = English

### 2.4. Treatment

All participants received 40 hours of SFT in either English or Spanish. Treatment was delivered during two-hour sessions, twice per week, for a total of 10 weeks by a trained bilingual clinician or research assistant. The treatment protocol, based on previous work in bilingual aphasia rehabilitation (Edmonds & Kiran, 2006; Kiran & Iakupova, 2011; Kiran & Roberts, 2010; Kiran et al., 2013), consisted of six steps that emphasized the semantic features of words to promote word retrieval (see Supplementary Table S3). Requirements for delivery and a list of modifications and accommodations to facilitate access to treatment have been reported elsewhere (Peñaloza et al., 2021; Scimeca et al., 2022).

#### 2.4.1. Stimuli

Stimuli were identified prior to the start of treatment based on performance on a large bilingual naming screener previously developed in our laboratory. The screener, which includes 273 colored pictured words from 13 semantic categories (e.g., fruits) with validated semantic features (Sandberg et al., 2020), was administered to all participants in both languages and responses were transcribed and scored by trained clinicians. Assessment language was counterbalanced across participants; therefore, some completed the screener in Spanish first and others, English. The screener could be broken up and administered across sessions if needed. Words were considered for treatment stimuli if they were named incorrectly in both English and Spanish. Six sets of stimuli were constructed for each participant that reflected their specific profile of naming difficulties. As reported in Scimeca et al. (2024), the first three sets contained words in the treated language (i.e., Spanish or English): set 1 consisted of trained words that were directly targeted in therapy (e.g., squirrel), set 2 consisted of semantically related words that were probed each week but were never explicitly trained (e.g., raccoon) and set 3 consisted of control words that were unrelated to the words in the previous sets, and which often came from different categories entirely (e.g., wrench). The remaining three sets consisted of direct translations of the words in sets 1–3 in the untreated language (e.g., *ardilla-mapache-llave inglesa*).

#### 2.4.2. Naming probes

Progress in treatment was evaluated using naming probes in each language that included 90 pictured words (15 words, in 3 sets, across the two languages). Probes were presented at the beginning of each testing or treatment session in language blocks (e.g., all English naming first) to minimize the possible effects of testing fatigue and cross-linguistic interference. Within each probe, the order of pictured words was randomized across sessions according to criteria described in Scimeca et al. (2024). Naming probes were self-paced and administered by bilingual clinicians who were instructed to transcribe all utterances from a participant including self-corrections, false starts and tangential comments elicited during naming. For each probe, instructions were provided in the target language; however, if during administration, participants began providing responses in the opposite language for any reason, no attempt was made by the clinician to repeat the instructions or encourage the participant to provide responses only in the target language. Correct responses included any appropriate label for a pictured item that was produced with no sound errors or one phoneme deviation from the expected target in the language being tested. When confronted with regional vocabulary, especially in Spanish, bilingual clinicians conferred with one another to establish consensus about the validity of certain responses. All other responses, including otherwise correct responses that were produced in the opposite (nontarget) language, were scored as incorrect. Incorrect responses were error-coded according to the scoring scheme presented in the following section.

### 2.5. Error scoring procedures

Naming responses were scored according to previously established criteria for error coding in bilingual aphasia (Kiran et al., 2014). These criteria provided guidance for analyzing responses in both the target language and the nontarget language, which is clinically important because prestroke language proficiency and poststroke language impairment may influence the degree to which participants retrieve words in either language. Each response received an error code between 1 and 10.5 corresponding to specific categories of word retrieval behaviors. On this scale: (i) all error codes are categorical; (ii) higher numerical values suggest responses that are closer to the target (e.g., a phonological error score of 8.5 suggests a response that is closer to the target than a neologism score of 2.5) and (iii) whole number scores generally denote responses in the nontarget language while scores ending in .5 generally denote responses in the target language. If participants produced more than one type of error within the same response, the error with the highest numerical value was coded. Table 3 provides a summary of the criteria for each error code with examples from the study participants in both English and Spanish.

**Table 3.** Error scoring criteria and examples in English and Spanish

		English		Spanish	
Error	Description	Target	Example	Target	Example
*No response in nontarget lang* (1)	No response, I do not know or similar in the language not being tested	wheat	no sé *I do not know*	casco *helmet*	sorry
*No response in target lang* (1.5)	No response, I do not know or similar in the language being tested	artichoke	I forgot it	lanza *spear*	no sé *I do not know*
*Neologism in nontarget lang* (2)	Nonword with less than 50% phonological overlap with target word in the language not being tested	crab	atuma	águila *eagle*	eliche
*Neologism in target lang* (2.5)	Nonword with less than 50% phonological overlap with target word in the language being tested	toaster	crostek	trinche *pitchfork*	wataca
*Perseveration to a nonprobe item* (3)	Repetition at least 3 times of a neologism or unrelated word not previously presented to participant	onion	love you, love you, love you	cola *tail*	lavado, lavado, lavado *washed*
*Perseveration to a probe in session* (3.5)	Repetition at least 3 times of a neologism or unrelated word that was previously presented to participant	toaster	camisa (prev. presented) *shirt*	ventana *window*	carme [carne] (prev. presented) *meat*
*Unrelated word in nontarget lang* (4)	Response that is semantically and phonologically unrelated to target in language not being tested	beetle	nabo *turnip*	imán *magnet*	porch
*Unrelated word in target lang* (4.5)	Response that is semantically and phonologically unrelated to target in language being tested	cranberry	brushes	somorgujo *loon*	polea *pulley*
*Circumlocution in nontarget lang* (5)	Word or utterance that contains a semantic description of the target in the language not being tested	sink	para manos *for hands*	granero *barn*	a building
*Circumlocution in target lang* (5.5)	Word or utterance that contains a semantic description of the target in the language being tested	apron	you put it on to cook	tostadora *toaster*	una maquina para que caliente *a machine for heating*
*Semantic error in nontarget lang* (6)	Word substitution or semantic paraphasia in the language not being tested	bench	silla *chair*	eneldo *dill*	parsley
*Semantic error in target lang* (6.5)	Word substitution or semantic paraphasia in the language being tested	saw	hammer	nabo *turnip*	cebolla *onion*
*Mixed error in nontarget lang* (7)	Word error that combines criteria from at least 2 other error categories in the language not being tested	clog	zancos [zuecos] *stilts*	maracuyá *passionfruit*	pamagrate [pomegranate]
*Mixed error in target lang* (7.5)	Word error that combines criteria from at least 2 other error categories in the language being tested	badger	accoon [racoon]	banco *stool*	stant [estante] *bookshelf*
*Phonological error in nontarget lang* (8)	Word error with at least 2 phonological deviations from the target in the language not being tested	skateboard	baneta [patineta] *skateboard*	maleta *suitcase*	cucase [suitcase]
*Phonological error in target lang* (8.5)	Word error with at least 2 phonological deviations from the target in the language being tested	skunk	skum	lobo *wolf*	ono
*Correct in nontarget lang* (9)	Correct translation equivalent, or 1 phoneme deviation, in the language not being tested	pomegranate	granada *pomegranate*	almohada *pillow*	pillow
*Motor speech but intelligible response* (9.5)	Otherwise correct response with noticeable dysarthic or apractic distortion	blueberry	booberry	dedal *thimble*	dedad
*Accent-influence response* (10)	Otherwise correct response containing minimal phonology of the other language	thumb, smoke	thomb, ehsmoke	collar *necklace*	colar [kolar]
*Correct in target lang* (10.5)	Correct response, or 1 phoneme deviation, in the language being tested	tweezers	weezers	chiltoma *bell pepper*	siltoma [nonword]

*Note.* Errors could occur in either language. Examples are presented based on responses produced when testing a given language. For example, a score of 3.5 was given to a response of camisa even though it is a Spanish word because it was produced repetitively in response to the English target “toaster”. Brackets [] indicate clinical judgment of participant’s word retrieval attempt. Responses in italics indicate English glosses for Spanish words.

### 2.6. Error scoring reliability

To assess error scoring reliability, two independent raters received 6 hours of training in error scoring by the first and fifth authors. After training, the two raters completed a calibration session in which they scored three full naming probes independently and received feedback to resolve discrepancies. Then, the raters scored 10% of all probes originally scored by the first author to determine interrater reliability (IRR) of the error scoring framework. The probes were chosen pseudorandomly so that all (i) participants had at least one session scored during training or independent scoring and (ii) all possible timepoints were represented at least five times. To quantify IRR, we calculated linear-weighted Cohen’s Kappa to assess categorical agreement – accounting for chance – and to reflect the ordinal nature of the error coding system. Results indicated near-perfect agreement across error categories with *κ =* 0.90 between the first author and rater 1 and *κ =* 0.89 between the first author and rater 2 (Hallgren, 2012; Landis & Koch, 1977).

### 2.7. Data analysis

For research question 1, we reexamined naming improvement in the treated and untreated language following procedures outlined in Scimeca et al. (2024). In the current study, we added data for semantically related words (to analyze outcomes across six word sets) and 14 more participants (*n* = 48 BWA). In both the treated and untreated language, we predicted the likelihood of a correct naming response across the six word sets via logistic mixed-effects regression. The models for outcomes in both languages were constructed with a combination of fixed and random effects informed by study design (Barr et al., 2013; Jaeger, 2008). We replicated the results of Scimeca et al. (2024) using the same model structures to investigate overall treatment outcomes. Therefore, fixed effects were proposed to include an interaction between probe session (continuous 0–15; 0–2 = baseline naming, 3–12 = treatment naming, 13–15 = posttreatment naming) and word set (categorical; sets 1–3), anomia severity at baseline (continuous *z*-score from the BNT in treated or untreated language) and a treatment language variable (categorical; 1 = treated in L1, 0 = treated in L2). During model fitting, the treatment language variable did not improve model fit in the treated (χ^2^(1) = 0.002, *p* = .958) or untreated (χ^2^(1) = 1.21, *p* = .270) languages and the term was removed from the final models. Random effects included intercepts for participant and item to capture differences in anomia severity at baseline according to these grouping factors as well as a by-participant slope for probe session to allow for various rates of improvement among the participants.

For research question 2, which focused on relationships between baseline error proportions and naming improvement, we fit six individual logistic mixed-effects regressions for each word set in each language with a fixed effect of probe session and the same random effects as outlined above. Next, we extracted individual effect size measures for improvement over time – defined as the by-participant random slope estimates for the session variable – from each of the six logistic mixed effects models using the coef() function in R (v4.0.2; R Core Team, 2020). This produced one estimate of improvement for each participant for each word set. Then, we computed the proportion of errors produced across each word set during each session by combining occurrences in the target and nontarget language for each error type and comparing the counts against the total number of errors. Error proportions were calculated for all error types except motor speech errors and accent responses given their low occurrence across participants. Lastly, we computed Spearman correlations between the error proportions and the individual effect sizes from each set to investigate relationships between anomia severity at baseline and eventual naming recovery for all 48 BWA. All correlation *p*-values were adjusted for multiple comparisons within word sets using the false-detection rate *q*-value of .05.

To address research question 3 (change in error rates), we employed negative binomial regression models to estimate the counts of error types and any changes in error production over the course of treatment. Negative binomial regression is a specialized case of Poisson regression for nonnegative count or rate data (Ver Hoef & Boveng, 2007). Whereas Poisson regression assumes the conditional mean and variance of the data are equivalent, negative binomial regression loosens this restriction and is better suited for data in which the variance exceeds the mean (i.e., overdispersion; Beaujean & Grant, 2019). Overdispersion may be more common when the underlying data contain many 0s (i.e., no counts observed) or when higher count values may be expected for certain combinations of predictors, which are both applicable to the error data in this study. We fit negative binomial regression models for each word set in each language as in research question 2. For each model, we compared the conditional means and variances and performed a likelihood ratio test between a Poisson regression and the negative binomial regression; for each of the six word sets, the negative binomial models fit the data significantly better. Fixed effects in the models included an interaction between probe session and error type (categorical; levels denote all error categories). Due to convergence issues, we did not include any random effects. Finally, we used contrast matrices – as outlined in Gilmore et al. (2022) – to extract individual intercepts and slopes by error category from each of the models to determine independent rates of change for all error types across the word set. Error category-specific model intercept and slope estimates from these analyses are reported in Supplementary Table S4. All intercept and slope tests for significance were also adjusted for multiple comparisons within word sets using the false-detection rate *q*-value of .05.

All data cleaning, modeling, and visualization procedures across research questions 1–3 were conducted in R using the following packages: lme4 (v1.1–23; Bates et al., 2015), tidyverse (v1.3.0; Wickham et al., 2019), broom (v0.7.6; Robinson et al., 2021), MASS (v7.3–51.6; Venables & Ripley, 2002), multcomp (v1.4–16; Hothorn et al., 2008) and cowplot (v1.1.0; Wilke, 2020).

## 3. Results

### 3.1. Naming improvement

#### 3.1.1. Naming improvement in the treated language

**Figure 2.**
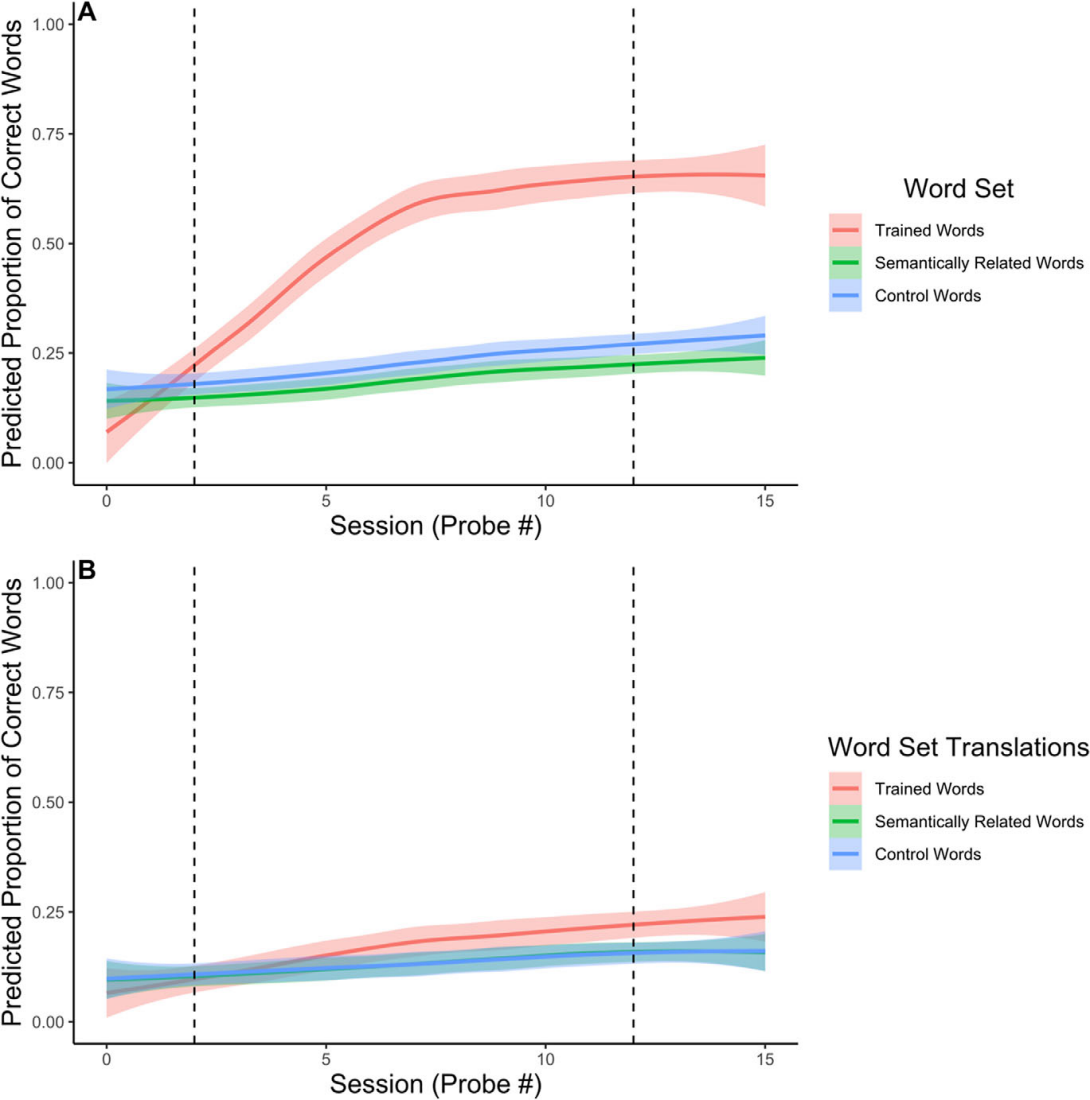
The predicted proportion of correct items across sets 1–3 is presented. The x-axis lists probe session number (0–2 = baseline, 3–12 = treatment, 13–15 = posttreatment). The y-axis shows predicted proportion values over time. The hashed vertical lines demonstrate divisions between study phases. (A) presents outcomes for the treated language and (B) presents outcomes for the untreated language. Shading represents the standard error for the predictions in each curve.

Consistent with the results of Scimeca et al. (2024), participants demonstrated significant naming improvement for trained words (set 1) in the treated language over the course of the intervention relative to control words (*β* = 0.25, SE = 0.009, *z* = 25.72, *p* < .001). However, there was no significant effect of within-language generalization – defined as a larger increase in improvement for semantically related words compared to control words (set 2; *β* = −0.01, SE = 0.009, *z* = −1.59, *p* = .110) nor significant improvement in control words alone (set 3; *β* = 0.03, SE = 0.02, *z* = 1.73, *p* = .082). As in Scimeca et al. (2024), participants with lower anomia severity at baseline showed greater naming improvement (*β* = 1.90, SE = 0.30, *z* = 6.26, *p* < .001). Predicted rates of improvement across word sets in the treated language are presented in Figure 2A and model outcomes are available in Table 4.

**Table 4.** Model results for overall accuracy in the treated and untreated language

			Random effects (variance)			Fixed effects				
Model	Syntax	Dependent variable	Intercept: item	Intercept: participant	Slope: session x participant	Predictor	*β*	SE	*z*-stat	*p*-value
1. Overall accuracy in the treated language	Score ~ session × Set + BNT-Score + (1|Item) + (Session|Participant)	Binary accuracy for items in the treated language	4.81	2.68	0.01	**Intercept**	**−3.21**	**0.29**	**−11.44**	**<.001**
						Session	0.03	0.02	1.73	.082
						**Set 1**	**0.70**	**0.09**	**7.46**	**<.001**
						Set 2	−0.08	0.10	−0.80	.419
						**Session** × **Set 1**	**0.25**	**0.009**	**25.72**	**<.001**
						Session × Set 2	−0.01	0.009	−1.59	.110
						**BNT-Score**	**1.90**	**0.30**	**6.26**	**<.001**
2. Overall accuracy in the untreated language	Score ~ session × Set + BNT-Score + (1|Item) + (Session|Participant)	Binary accuracy for items in the untreated language	6.43	3.97	0.01	**Intercept**	**−5.62**	**0.33**	**−15.82**	**<.001**
						**Session**	**0.04**	**0.02**	**2.24**	**.024**
						**Set 1**	**0.42**	**0.13**	**3.18**	**.001**
						**Set 2**	**0.42**	**0.13**	**3.11**	**.001**
						**Session** × **Set 1**	**0.09**	**0.01**	**7.68**	**<.001**
						Session × Set 2	0.00	0.01	0.02	.981
						**BNT-Score**	**3.05**	**0.35**	**8.53**	**<.001**

*Note.* Random intercepts for item and participant capture variance arising from these factors during baseline naming. The by-participant random slope for session allows for varying rates of improvement across participants; *Session* = a continuous predictor with values 0–15; *Set* = a categorical predictor with three levels corresponding to word set (i.e., 1 and 2; reference level = 3); *BNT Score* = a continuous predictor based on the distribution of *z*-scored BNT performance among the participants in either the treated or untreated language; *β* = the log odds coefficient; *SE* = Standard Error. Bolded predictors are significant at *p* < .05. The estimate for *Session* in each model represents the log odds for set 3 words over time.

#### 3.1.2. Naming improvement in the untreated language

Over the course of the intervention, there was a significant effect of cross-language generalization to translations of trained words (*β* = 0.09, SE = 0.01, *z* = 7.68, *p* < .001), relative to control words, consistent with Scimeca et al. (2024). This cross-language effect for trained words was greater than the rate of improvement for translations of control words alone (set 3; *β* = 0.04, SE = 0.02, *z* = 2.24, *p* = .024). As in the treated language, there was no significant cross-language improvement for semantically related words above control words (*β* = 0.00, SE = 0.01, *z* = 0.02, *p* = .981). Finally, participants with lower anomia severity at baseline in the untreated language demonstrated greater naming improvement across sets (*β* = 3.05, SE = 0.35, *z* = 8.53, *p* < .001), consistent with Scimeca et al. (2024). The rates of improvement across untreated sets 1–3 are presented in Figure 2B and model outcomes are available in Table 4.

### 3.2. Baseline error proportions and naming improvement

**Figure 3.**
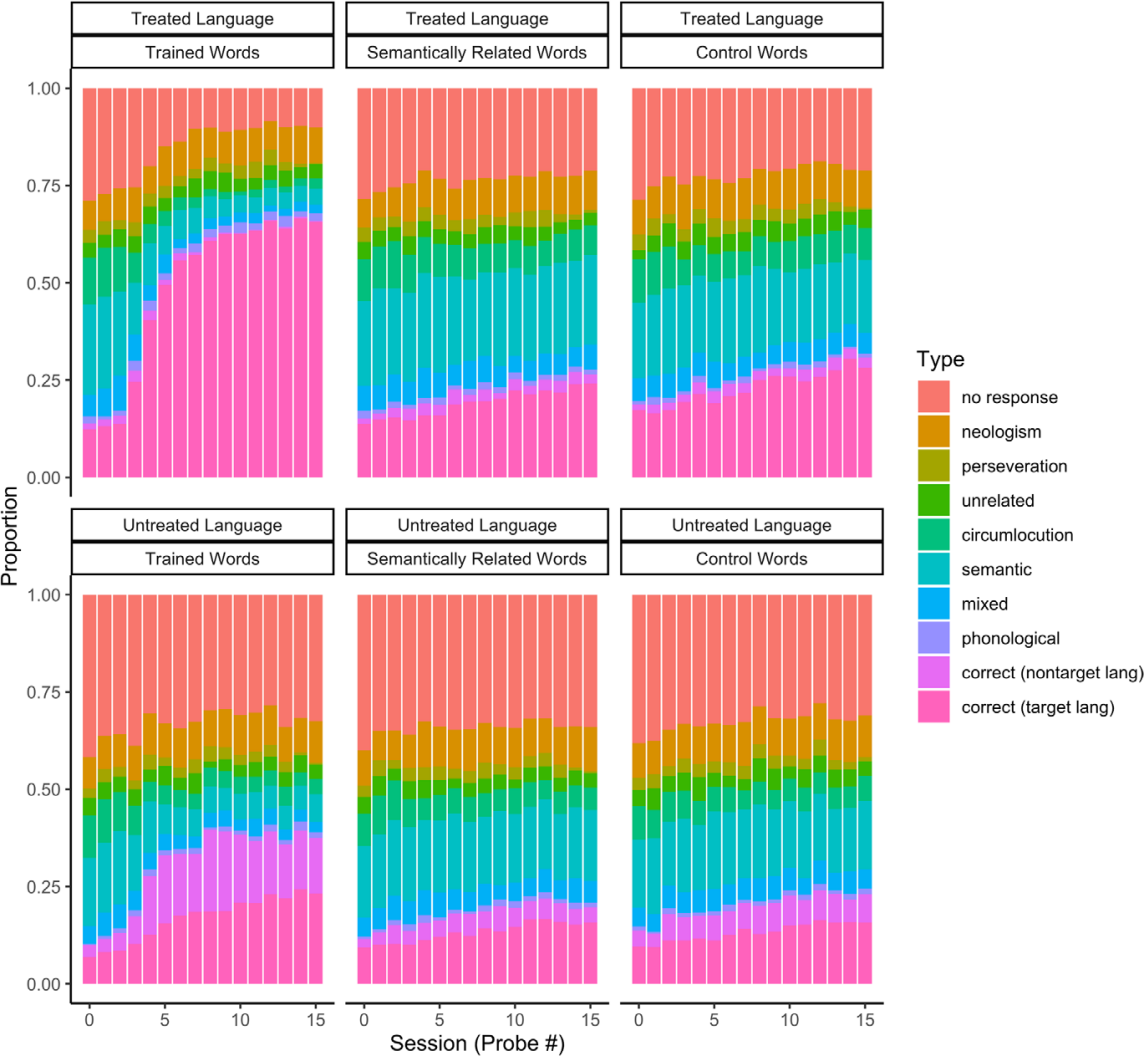
Response proportions are shown for each word set in each language. Treated language sets are shown in the top panels and untreated language sets are shown in the bottom panels. The x-axis lists the probe session number to demonstrate how proportions of each response (y-axis) change across intervention. Accent and motor responses were excluded from analysis given their low occurrence across word sets.

Error proportions for the six word sets across the treated and untreated languages are reported in Figure 3.

#### 3.2.1. Relationship between error proportions and improvement in the treated language

For trained words, five sets of baseline error correlations emerged to characterize participant-specific recovery (Table 5; treated set 1). Lower proportions of neologisms (*ρ* = −0.39, *q* = .011), perseverations (*ρ* = −0.26, *q* = .027) and unrelated word errors (*ρ* = −0.46, *q* = .001) were weakly to moderately associated with improved naming accuracy over time. Additionally, a higher proportion of circumlocution (*ρ* = 0.52, *q* < .001) and semantic errors (*ρ* = 0.5, *q* < .001) were moderately associated with greater naming improvement, suggesting that predominantly lexical-semantic errors at baseline may be indicative of better response to SFT for trained words. By contrast, there were no significant associations between error type proportions and individual-specific rates of improvement for semantically related words or control words (Table 5; treated sets 2 and 3, respectively).

**Table 5.** Spearman correlations between error type proportions at baseline and individual effect sizes

Error type	Treated set 1 ES	Treated set 2 ES	Treated set 3 ES
*No response*	*ρ* = −0.06, *p* = .670, *q* = .670	*ρ* = 0.10, *p* = .491, *q* = .714	*ρ* = 0.22, *p* = .123, *q* = .557
*Neologism*	***ρ* = −0.39, *p* = .005, *q* = .011**	*ρ* = −0.08, *p* = .555, *q* = 714	*ρ* = −0.11, *p* = .425, *q* = .636
*Perseveration*	***ρ* = −0.26, *p* = .015, *q* = .027**	*ρ* = −0.19, *p* = .183, *q* = .665	*ρ* = −0.25, *p* = .076, *q* = .557
*Unrelated*	***ρ* = −0.46, *p* < .001, *q* = .001**	*ρ* = −0.05, *p* = .687, *q* = .773	*ρ* = −0.13, *p* = .349, *q* = .628
*Circumlocution*	***ρ* = 0.52, *p* < .001, *q* < .001**	*ρ* = −0.03, *p* = .826, *q* = .826	*ρ* = −0.10, *p* = .495, *q* = .636
*Semantic*	***ρ* = 0.5, *p* < .001, *q* < .001**	*ρ* = 0.13, *p* = .363, *q* = .665	*ρ* = 0.15, *p* = .279, *q* = .628
*Mixed*	*ρ* = 0.23, *p* = .050, *q* = .075	*ρ* = 0.24, *p* = .088, *q* = .665	*ρ* = 0.07, *p* = .599, *q* = .674
*Phonological*	*ρ* = 0.064, *p* = .638, *q* = .670	*ρ* = 0.13, *p* = .369, *q* = .665	*ρ* = −0.04, *p* = .773, *q* = .773
*Correct (nontarget)*	*ρ* = 0.14, *p* = .147, *q* = .189	*ρ* = 0.15, *p* = .293, *q* = .665	*ρ* = 0.14, *p* = .341, *q* = .628

Error type	Untreated Set 1 ES	Untreated Set 2 ES	Untreated Set 3 ES
*No response*	*ρ* = −0.18, *p* = .198, *q* = .316	*ρ* = 0.008, *p* = .953, *q* = .953	*ρ* = 0.04, *p* = .779, *q* = .792
*Neologism*	*ρ* = −0.25, *p* = .076, *q* = .172	*ρ* = −0.19, *p* = .181, *q* = .347	*ρ* = 0.03, *p* = .792, *q* = .792
*Perseveration*	*ρ* = −0.06, *p* = .644, *q* = .644	*ρ* = −0.19, *p* = .193, *q* = .347	*ρ* = −0.10, *p* = .459, *q* = .792
*Unrelated*	*ρ* = −0.15, *p* = .278, *q* = .357	*ρ* = −0.08, *p* = .562, *q* = .804	*ρ* = −0.04, *p* = .774, *q* = .792
*Circumlocution*	*ρ* = 0.28, *p* = .046*, *q* = .139	*ρ* = 0.22, *p* = .127, *q* = .347	*ρ* = 0.17, *p* = .243, *q* = .548
*Semantic*	***ρ* = 0.39, *p* = .005, *q* = .046**	*ρ* = 0.24, *p* = .087, *q* = .347	*ρ* = 0.17, *p* = .237, *q* = .548
*Mixed*	***ρ* = 0.37, *p* = .010, *q* = .046**	*ρ* = 0.22, *p* = .122, *q* = .347	*ρ* = 0.17, *p* = .243, *q* = 548
*Phonological*	*ρ* = 0.18, *p* = .211, *q* = .316	*ρ* = 0.03, *p* = .795, *q* = .894	*ρ* = 0.04, *p* = .781, *q* = .792
*Correct (nontarget)*	*ρ* = −0.08, *p* = .549, *q* = .618	*ρ* = 0.07, *p* = .625, *q* = 804	*ρ* = 0.33, *p* = .018*, *q* = .166

*Note. Set 1* = Trained Items; *Set 2* = Semantically Related Items; *Set 3* = Control Items; *ES* = Individual effect size measures of naming accuracy within each set. Bolded Spearman correlations survived FDR-correction at *q* < .05. Asterisks denote *p*-values which are significant at alpha <.05.

#### 3.2.2. Relationship between error proportions and improvement in the untreated language

For trained translations (Table 5; untreated set 1), higher proportions of semantic errors (*ρ* = 0.39, *q* = .046) and mixed errors (*ρ* = 0.37, *q* = .046) at baseline were weakly associated with greater naming improvement across participants. This pattern of baseline semantic errors was similarly observed for trained words in the treated language, further suggesting a relationship between some preserved lexical-semantic access at baseline and eventual naming improvement in the context of SFT. For semantically related and control translations (Table 5; untreated sets 2 and 3), any significant associations between error types and naming improvement did not survive correction.

### 3.3. Change in naming errors

The results of the contrast matrices for the negative binomial regression models for each item set are reported in Supplementary Table S4.

#### 3.3.1. Naming errors in the treated language

**Figure 4.**
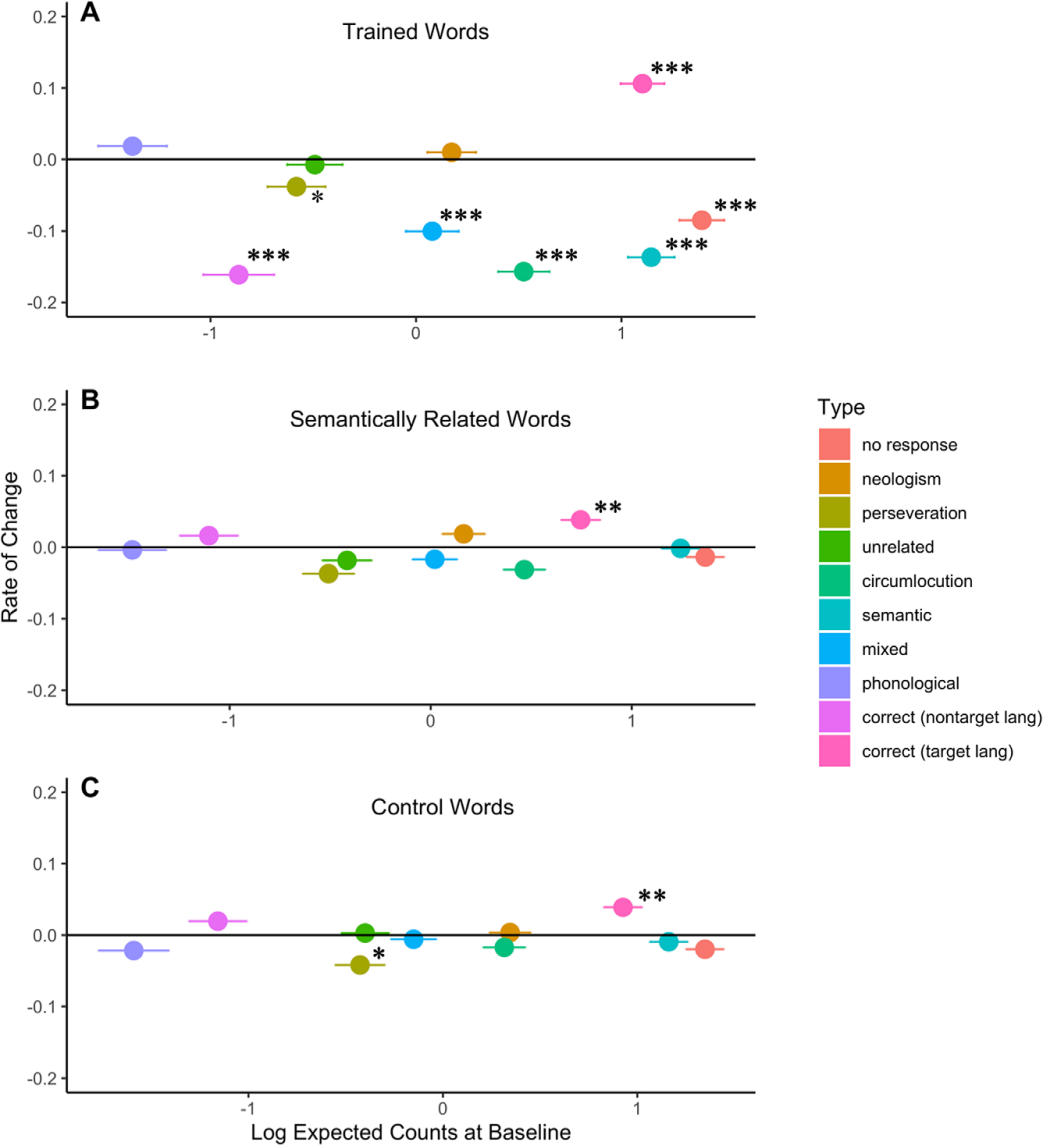
The x-axis represents the log of the expected number of each error type at the first baseline probe in the treated language; toward −1 represents fewer occurrences of an error type and toward 1 indicates more occurrences of an error type. The y-axis represents the log of the expected change in the number of each error over time (Rate of Change); values above 0 indicate an increase in an error type while values below 0 indicate a decrease in an error type. Rates of change for each error type are calculated independently of one another. Asterisks denote significant rates of change after multiple comparison correction. A) shows error rates for trained items; B) shows error rates for semantically related items; C) shows error rates for control items.

For trained words in the treated language, a variety of error types significantly decreased over time – consistent with a robust treatment effect and a large increase in the number of responses that were correct in the target language (*β* = 0.10, SE = 0.01, *z* = 8.85, *q* < .001). No response (*β* = −0.08, SE = 0.01, *z* = −6.61, *q* < .001), perseveration (*β* = −0.03, SE = 0.01, *z* = −2.27, *q* = .033), circumlocution (*β* = −0.15, SE = 0.01, *z* = −9.33, *q* < .001), semantic (*β* = −0.13, SE = 0.01, *z* = −9.63, *q* < .001), mixed (*β* = −0.10, SE = 0.01, *z* = −6.13, *q* < .001), and correct in the nontarget language (*β* = −0.16, SE = 0.02, *z* = −6.12, *q* < .001) responses all significantly decreased over time and these changes survived correction. These effects (treated set 1) are well represented in Figure 4A, which shows the relative occurrence of each error at baseline (i.e., further to the left means fewer errors) and the rate of change (i.e., more positive change in the upper half of the plot means an increase).

For semantically related words, there was a significant increase in the number of correct responses in the target language (*β* = 0.03, SE = 0.01, *z* = 3.49, *q* < .01). The number of correct responses was much smaller than for trained words – consistent with overall naming improvement. No other relationships survived correction. Baseline errors and rates of change for semantically related words (treated set 2) are presented in Figure 4B.

For control words, a significant increase in the number of correct responses in the target language (*β* = 0.03, SE = 0.01, *z* = 3.44, *q* < .01) co-occurred with a significant decrease in the number of perseverations (*β* = −0.04, SE = 0.01, *z* = −2.68, *q* = .036). Baseline errors and rates of change for control words (treated set 3) are presented in Figure 4C.

#### 3.3.2. Naming errors in the untreated language

**Figure 5.**
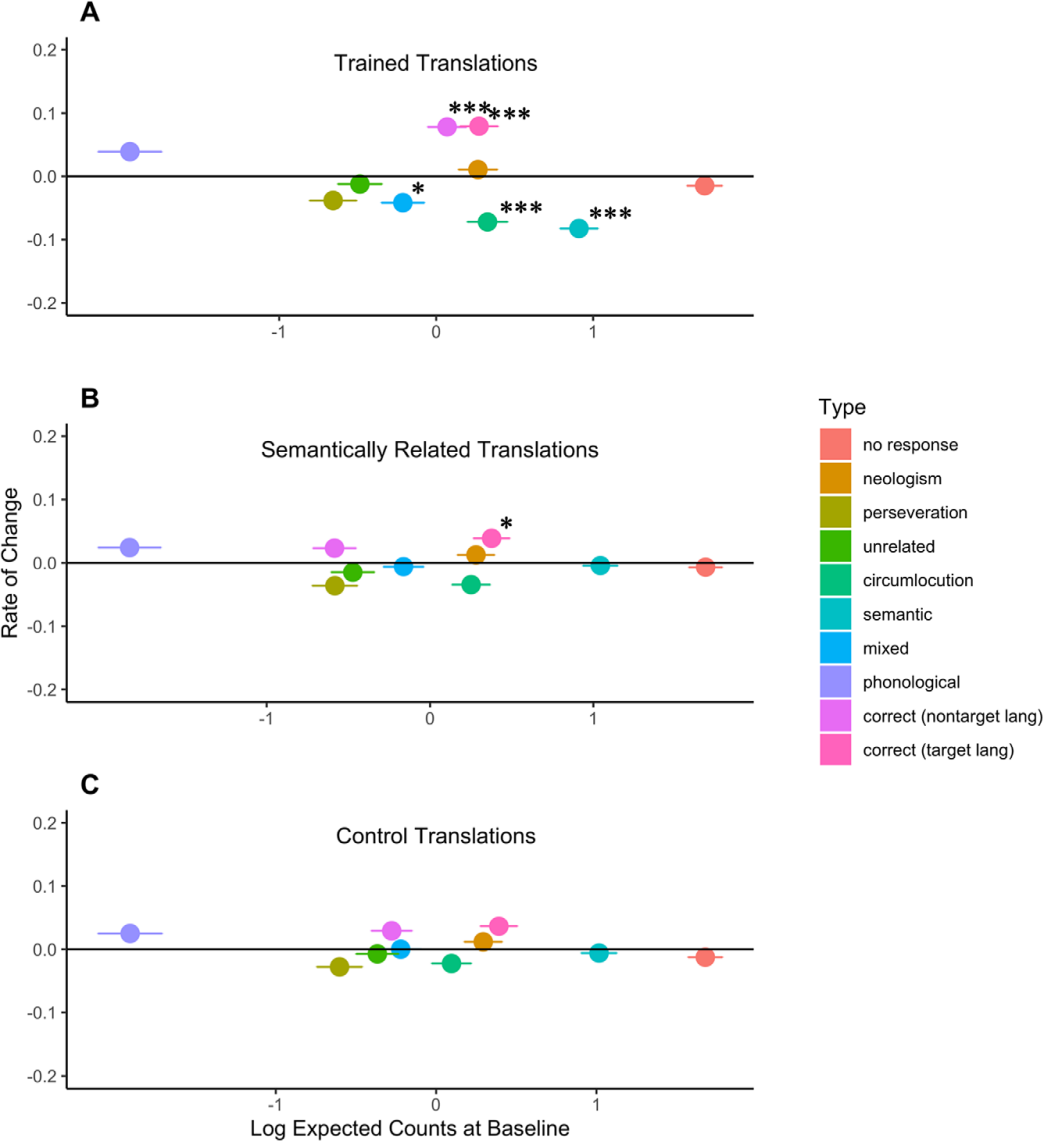
The x-axis represents the log of the expected number of each error type at the first baseline probe in the untreated language; toward −1 represents fewer occurrences of an error type and toward 1 indicates more occurrences of an error type. The y-axis represents the log of the expected change in the number of each error over time (rate of change); values above 0 indicate an increase in an error type while values below 0 indicate a decrease in an error type. Rates of change for each error type are calculated independently of one another. Asterisks denote significant rates of change after multiple comparison correction. A) shows error rates for trained translations; B) shows error rates for semantically related translations; C) shows error rates for control translations.

For trained translations in the untreated language, participants provided significantly more correct responses in both the target (*β* = 0.07, SE = 0.01, *z* = 5.82, *q* < .001) and nontarget language (*β* = 0.07, SE = 0.01, *z* = 5.64, *q* < .001) over time. The comparable strength of these improvement effects suggests that participants more consistently accessed the correct lexical-semantic representations for trained translations over time, but they may have produced the word in the target language inconsistently. Participants also produced fewer circumlocution (*β* = −0.07, SE = 0.01, *z* = −4.65, *q* < .001), semantic (*β* = −0.08, SE = 0.01, *z* = −5.72, *q* < .001) and mixed (*β* = −0.04, SE = 0.01, *z* = −2.55, *q* = .021) errors. These effects (untreated set 1) are well represented in Figure 5A, which shows the baseline rate of each error type and the associated change in production over time.

Error patterns for translations of semantically related words were in line with responses for semantically related words in the treated language. Participants produced significantly more correct responses in the target language (*β* = 0.03, SE = 0.01, *z* = 3.06, *q* < .05). All error shifts for translations of semantically related words (untreated set 2) are presented in Figure 5B.

Finally, there were no significant error shifts for translations of control items (untreated set 3; see Figure 5C below).

## 4. Discussion

The current study examined patterns of naming improvement for a large cohort of BWA who received SFT in either Spanish or English. Specifically, we investigated patterns of improvement over time for word sets in the treated and untreated language. Then, we performed error analyses to determine i) whether a relationship existed between types of responses at baseline and eventual rates of improvement and ii) how the evolution of error types over time might capture naming improvement beyond a simple binary scoring system (i.e., correct/incorrect). Broadly, we showed a robust treatment effect for trained words and smaller cross-language generalization effects to (i) translations of trained words and (ii) translations of control words in the untreated language. No patterns of generalization to semantically related words in either language or to control words in the treated language were observed. Error analyses in the treated language uncovered various relationships between the proportions of errors at baseline and rates of improvement as well as changes in error counts over the course of treatment – most of which were observed for trained words rather than semantically related or control words. In the untreated language, relationships between the proportions of errors and naming improvement and changes in error counts were observed mostly for translations of trained words.

### 4.1. Naming improvement and baseline error proportions in the treated language

First, we observed a large treatment effect in which the likelihood of a correct response for trained words significantly increased over time relative to control words, which replicated the findings using the participant sample from Scimeca et al. (2024). Second, although Figure 2A showed slight increases in accuracy for both semantically related and control words, there was no statistically significant effect of within-language generalization – semantically related words were not higher in accuracy compared to control words (see Table 4). Overall, these results underscore the efficacy of word retrieval interventions for trained words in BWA (Edmonds & Kiran, 2006; Kiran et al., 2013, Scimeca et al., 2024), and pattern with other studies that have observed much larger direct treatment effects than within-language generalization effects (Goral et al., 2023; Lee & Faroqi-Shah, 2024). However, further analysis is needed to determine why we did not observe within-language generalization at the group level in our data. In addition to the naming improvement effects, we found that participants with less severe anomia in the treated language at baseline demonstrated a higher likelihood of correct responses across word sets. This finding is consistent with recent work demonstrating that baseline language abilities are informative predictors of response to treatment in chronic aphasia (Braun & Kiran, 2022; Quique et al., 2019; Scimeca et al., 2024).

The subsequent analyses of errors at baseline revealed significant correlations between higher proportions of circumlocution and semantic errors and lower proportions of neologisms, perseverations and unrelated errors at baseline and individual rates of naming improvement for trained words in the treated language (see Table 5). In Figure 1, we proposed that unrelated errors, circumlocutions and semantic errors represented distorted semantic access; within this group, however, circumlocutions and semantic errors have some semantic overlap with a given target word. Unrelated errors have comparatively fewer semantic features in common with a target, and this may explain why higher proportions of circumlocutions and semantic errors but *lower* proportions of unrelated errors at baseline were associated with greater naming improvement. These findings suggest that participants who demonstrated more intact lexical-semantic processing skills during picture naming in both the target and nontarget languages – that is, they showed evidence of semantic access, but incomplete or incorrect lexical selection on a gradient (i.e., unrelated<circumlocution = semantic) – tended to show greater naming improvement. Likewise, participants who produced fewer neologisms and perseverations that, under some lexical-semantic processing accounts, are hypothesized to occur before semantic access to the target word form (e.g., Kiran et al., 2014), tended to perform better in therapy. Taken together, these findings may provide information about anomia severity like formal assessments of naming and are consistent with our overall hypotheses in Section 1.3.

We did not find any relationships between error proportions at baseline and rates of improvement for semantically related words or control words in the treated language. These null effects may suggest that naming behavior was similar for both types of words because their error patterns were unremarkable.

### 4.2. Naming improvement and baseline error proportions in the untreated language

For naming improvement in the untreated language, we observed a moderate and statistically significant cross-language generalization effect for translations of trained words in which the likelihood of a correct response increased over time relative to translations of control words. This finding supports other studies that have observed cross-language effects in bilingual treatment (Croft et al., 2011; Edmonds & Kiran, 2006; Kiran et al., 2013; Kiran & Iakupova, 2011; Kiran & Roberts, 2010; Peñaloza et al., 2021; Scimeca et al., 2024). Figure 2B demonstrates that the accuracy for translations of semantically related and control words both rose slightly over time; however, only the control word effect was statistically significant. Like the null finding for within-language generalization, we did not find any significant cross-language generalization to semantically related translations (see Table 4). Taken all together, these findings suggest that generalization to trained translations occurred, given the direct treatment effect in the treated language mediated by increased activation throughout the semantic network flowing to translation equivalents (Costa et al., 2000). Improvement in control translations cannot be explained this way since treated control words did not improve; nevertheless, one possible explanation could be that increased generalized activation throughout the system from treatment benefited control translations. However, further analysis is needed to determine why these untreated words improved while semantically related translations did not. Additionally, our finding that lower anomia severity at baseline in the untreated language predicted better naming improvement extends the findings from Scimeca et al. (2024) and is again in line with other studies (e.g., Braun & Kiran, 2022; Quique et al., 2019) that have reported relationships between measures of impairment and subsequent recovery. These findings support the notion that more intact lexical-semantic processing in both an untreated and treated language leads to better performance in anomia therapy.

Like trained words, greater naming improvement for trained translations was associated with higher proportions of semantic errors – and uniquely – mixed errors at baseline. However, we did not find the same relationship between lower proportions of neologisms, perseverations and unrelated errors and eventual naming improvement. Overall, these findings further suggest that some evidence of intact lexical-semantic processing at baseline in both languages may lead not only to better direct treatment response – but in the case of BWA – also to greater cross-language generalization to translations of trained items. In this manner, the results mirror those of Scimeca et al. (2024), which showed that anomia severity at baseline (as determined by performance on the BNT) predicted both direct treatment effects and cross-language generalization effects. In terms of error analysis, these results indicate that even before intervention begins, participants’ responses may shed light on their capacity for change in both a treated and untreated language at the end of treatment.

### 4.3. Changes in error patterns in the treated language

For trained words in the treated language, we observed significant decreases in the expected number of no response, perseveration, circumlocution, semantic, mixed and nontarget language (correct) errors with concurrent increases in the correct responses in the target language (see Figure 4 and Supplementary Table S4). Overall, it is likely participants experienced increased semantic activation from SFT that resulted in more accurate lexical retrieval and subsequent phonological retrieval. What is striking about these results is that even more severe errors, such as no responses and perseverations, decreased over time in addition to errors that belied some level of semantic access (e.g., circumlocutions and semantic errors). At one level, these findings suggest that the SFT administered was effective in rehabilitating a variety of error types to improve naming recovery. These results are also in line with several previous error analysis studies that found fewer omission errors for trained words after word retrieval treatment (Abel et al., 2007; Edmonds & Kiran, 2006; Kendall et al., 2013; Minkina et al., 2016). The current study is the first to replicate this finding with BWA using time-series data.

For semantically related words in the treated language, we observed a significant increase in the number of correct responses over time, but no other changes that survived correction. For control words, only perseveration responses decreased significantly, and correct responses in the target language increased significantly. Small but significant increases in the total number of correct responses for semantically related and control words pattern with the results from Lee and Faroqi-Shah (2024), which found small within-language generalization effects to related and unrelated words across bilingual word retrieval studies. Additionally, decreases in perseveration errors were observed for one bilingual participant in Kurland and Falcon (2011) following word retrieval therapy. However, Kendall et al. (2013) found an increase in the number of mixed errors for untrained words in their study, indicating increased phonological and semantic activation throughout the word retrieval cascade as a function of treatment. Although we did not find similar changes to error patterns for our untrained words, the decrease in perseverations for control words in the treated language suggests some movement toward less severe anomia, consistent with the interpretation of error patterns in Kendall et al. (2013) and Li et al. (2020).

### 4.4. Changes in error patterns in the untreated language

For translations of trained words in the untreated language, we observed significant increases in correct responses in *both* the target and nontarget languages, and again with significant decreases in circumlocution, semantic and mixed errors. What is striking about this result is that the positive rate of change for both types of correct responses is approximately the same (Figure 5A and Supplementary Table S4). These data suggest that direct feedback about word retrieval accuracy for trained words in the treated language supported some participants in retrieving the correct label in the untreated language. As shown in Figure 1, correct responses in the nontarget language constitute evidence for a cross-language generalization effect whereby increased lexical-semantic activation provided by SFT likely flowed along (i) the associative connections from the semantic-conceptual level and ii) the bidirectional connections between treated and untreated lexical representations (e.g., Kroll & Stewart, 1994). Furthermore, correct responses in the nontarget language suggest that some participants learned the correct label for a given picture (i.e., for a trained item in the treated language) and used that label when naming in the untreated language, perhaps because the activation threshold for this trained item was much lower than its translation equivalent. It is important to note that our interpretation of the cross-linguistic errors departs from the results of other bilingual studies – such as Li et al. (2020) – that ascribed an increase in cross-linguistic errors to deficits in language control and inhibition (Green, 1998). Other studies (Edmonds & Kiran, 2006; Keane & Kiran, 2015; Kurland & Falcon, 2011) also reported increases in cross-linguistic errors as a negative consequence of treatment in one language for BWA. One explanation for the difference between our study and others is that our scoring scheme allowed us to distinguish cross-linguistic responses that were otherwise correct from all other kinds of cross-linguistic responses. In these cases, we argue these responses may represent a word retrieval strategy that preserved communicative content even if the response was in the other, unexpected language, as other work has recently suggested (Mooijman et al., 2025; Peñaloza et al., 2025).

Shifts in error patterns for translations of semantically related words in the untreated language mirrored the patterns observed in the same set in the treated language (i.e., increase in correct target responses and no other changes). That these patterns were identical is further evidence that the BWA in our study co-activated lexical representations during naming in a language nonselective manner (Colomé, 2001; Costa et al., 1999; Costa, 2005; de Bot, 1992; Duyck et al., 2007; Kroll et al., 2006; La Heij, 2005; Libben & Titone, 2009).

### 4.5. Limitations and future directions

Some limitations of this work should be considered. To keep our analyses specific to our research questions, we mostly focused on relationships between time in intervention and changes in word retrieval errors. In reality, participant-level predictors such as bilingual language proficiency (Goral & Lerman, 2020) and psycholinguistic predictors associated with the treatment stimuli have been shown to affect response to treatment (Braun & Kiran, 2022; Scimeca et al., 2024). Future analyses that take these factors into account while conducting error analyses could improve our understanding of whether certain error types are influenced by factors other than those strictly related to the intervention.

Additionally, it should be acknowledged that the error analysis procedures in this study were time-consuming to complete, given that coding was completed by hand. Implementing these procedures in a clinical setting could be hampered by time constraints without some level of automation. In the future, studies in bilingual aphasia may investigate the feasibility of automated or partially automated scoring schemes to improve the availability and utility of error scoring data as has recently been demonstrated in monolingual aphasia (Ross et al., 2019). Future analyses may consider additional ways to examine error scoring data such as investigating differential patterns via participants who respond to treatment and those who do not (i.e., responder versus nonresponder analysis).

Finally, we observed a variety of i) relationships between baseline proportions of errors and naming improvement and ii) shifts in error patterns that did not survive correction in both the treated and untreated language. Some of these findings are worthy of further exploration either with new samples of BWA and/or other word retrieval treatments. For example, in addition to the increase in the number of correct responses in the target language, significant decreases were noted for perseveration and circumlocution errors for semantically related items in the treated language; however, these shifts did not survive correction. These patterns were different than the multiple category decreases observed for trained items, which could provide evidence that different types of errors are expected for words that are directly trained versus those that are untrained. Additionally, we considered perseveration responses to be more severe naming errors while circumlocutions were less severe. Given that both decreased in this word set, some error categories may be relatively invariant to the severity of anomia as Kendall et al. (2013) suggested in their work.

In another example, Figure 3 shows an increase (positive slope) in the number of correct responses in the target language for translations of control words over time; however, Supplementary Table S4 demonstrates this effect did not survive correction. Additionally, there was a rise (albeit nonsignificant) in the expected number of correct responses in the nontarget language for translations of semantically related and control words in the untreated language that also did not survive correction. For translations of trained words, we suggested that the increase in correct responses in the nontarget language represented a word retrieval strategy due to direct feedback about naming accuracy for trained words. However, this hypothesis cannot account for the increase in this error type in the other word sets, given that participants never received feedback about naming accuracy for sets 2 and 3 in the treated language. Therefore, it is possible that a rise in these types of errors constitutes cross-language interference in naming. Previous work in bilingual aphasia has noted increases in cross-language intrusions i) in the context of treatment-induced recovery (Edmonds & Kiran, 2006; and following therapy in L1 specifically in Keane & Kiran, 2015) and ii) for BWA with more severe anomia relative to those with milder anomia (Goral et al., 2019). Our results suggest that across participants, cross-language interference could arise when therapy is provided only in one language, but the conditions that might contribute to this cognitive-linguistic behavior may be more visible in future analyses that directly account for participant-specific factors.

## 5. Conclusions

In sum, this study further supports the efficacy of word retrieval therapy for BWA and shows that error analyses of word retrieval data may provide useful insight into shifts in behavior that may not be captured by traditional (i.e., binary) accuracy scoring. In clinical settings, BWA may demonstrate various patterns of treatment-induced word retrieval in both a treated and untreated language. Ultimately, some pattern shifts may suggest word retrieval strategies while others may reflect natural consequences of co-activation in the bilingual mental lexicon.

## Supporting information

Scimeca et al. supplementary material Scimeca et al. supplementary material

## Data Availability

The data and analyses that support the findings of this study are available from the authors upon request.

## Supplementary material

The supplementary material for this article can be found at http://doi.org/10.1017/S1366728925100370.
