# Supplementary material for: The evolution of word retrieval errors during semantic feature-based therapy in bilingual aphasia
Source: Biling (Camb Engl). 2025 Aug 11:1–18. Online ahead of print. doi: 10.1017/S1366728925100370 (PMC12520239; doi:10.1017/S1366728925100370)
Supplement: Scimeca et al. supplementary material [file S1366728925100370sup001.docx]

Supplementary Table S1. *Individual* *demographic and bilingual language characteristics for Spanish-English BWA*

| ID |  |  |  |  |  |  | L1 | | | | L2 | | | |
| --- | --- | --- | --- | --- | --- | --- | --- | --- | --- | --- | --- | --- | --- | --- |
|  |  |  | |  |  |  | Language  Use | | Language Ability Rating % | | Language  Use | | Language Ability Rating % | |
|  | Sex | Age | MPO | Edu | L1 | L2 AoA | Pre | Post | Pre | Post | Pre | Post | Pre | Post |
| P01 | M | 82.4 | 401.1 | 16 | S | 35 | 0.62 | NA | 1 | NA | 0.38 | NA | 0.8 | NA |
| P02 | F | 54.7 | 58.8 | 17 | S | 6 | 0.54 | 0.1 | 1 | 0.57 | 0.46 | 0.9 | 0.54 | 0.31 |
| P03 | M | 44.5 | 19.5 | 16 | S | 6 | 0.01 | 0.02 | 0.65 | 0.57 | 0.99 | 0.98 | 0.85 | 0.6 |
| P04 | F | 24.9 | 6.3 | 16 | S | 5 | 0.14 | 0.42 | 0.88 | 0.31 | 0.86 | 0.58 | 1 | 0.45 |
| P05 | F | 26.6 | 129.6 | 12 | S | 5 | 0.91 | 0.66 | 0.8 | 0.68 | 0.09 | 0.34 | 0.8 | 0.62 |
| P06 | M | 68.3 | 244.7 | 16 | S | 27 | 0.29 | 0.7 | 0.88 | 0.77 | 0.71 | 0.3 | 0.91 | 0.62 |
| P07 | F | 47.2 | 53.2 | 19 | S | 18 | 0.09 | 0.65 | 1 | 0.6 | 0.91 | 0.35 | 0.91 | 0.37 |
| P08 | M | 53.5 | 37.9 | 17 | S | 12 | 0.02 | 0.31 | 1 | 0.94 | 0.98 | 0.69 | 1 | 0.85 |
| P09 | M | 77.2 | 26.8 | 16 | S | 18 | 0.24 | 0.41 | 0.97 | 0.68 | 0.76 | 0.59 | 1 | 0.68 |
| P10 | F | 78.5 | 38.5 | 11 | S | 10 | 0.45 | 0.29 | 1 | 0.51 | 0.55 | 0.71 | 0.97 | 0.48 |
| P11 | M | 70.5 | 6 | 12 | S | 15 | 0.7 | 0.75 | 1 | 0.85 | 0.3 | 0.25 | 0.88 | 0.42 |
| P12 | F | 27.4 | 48.6 | 14 | S | 11 | 0.37 | 0.81 | 1 | 0.82 | 0.63 | 0.19 | 1 | 0.57 |
| P13 | F | 53.9 | 44.4 | 16 | E | 3 | 0.74 | 0.98 | 1 | 0.74 | 0.26 | 0.02 | 0.74 | 0.45 |
| P14 | M | 69.3 | 10.3 | 12 | S | 3 | 0.25 | 0.06 | 1 | 0.42 | 0.75 | 0.94 | 1 | 0.51 |
| P15 | F | 55.6 | 41 | 19 | E | 13 | 1 | 1 | 1 | 0.77 | 0 | 0 | 0.31 | 0.28 |
| P16 | M | 62.7 | 23.9 | 10 | S | 16 | 0.66 | 0.5 | 1 | 0.51 | 0.34 | 0.5 | 0.82 | 0.54 |
| P17 | F | 47.4 | 12.6 | 9 | S | 16 | 0.6 | 0.52 | 1 | 0.85 | 0.4 | 0.48 | 0.8 | 0.77 |
| P18 | M | 56.7 | 51.5 | 9 | S | 5 | 0.75 | 0.32 | 0.85 | 0.85 | 0.25 | 0.68 | 0.74 | 0.8 |
| P19 | M | 39.6 | 40.3 | 13 | S | 21 | 0.35 | 0.54 | 1 | 0.31 | 0.65 | 0.46 | 1 | 0.31 |
| P20 | M | 42.7 | 22.7 | 12 | E | 5 | 0.81 | 1 | 1 | 0.34 | 0.19 | 0 | 1 | 0.2 |
| P21 | M | 62.7 | 52.9 | 16 | S | 8 | 0.26 | 0.04 | 0.8 | 0.74 | 0.74 | 0.96 | 1 | 0.71 |
| P22 | F | 69.3 | 35 | 16 | S | 17 | 0.48 | NA | 1 | 0.37 | 0.52 | NA | 1 | 0.35 |
| P23 | F | 21.5 | 23.4 | 12 | S | 3 | 0.3 | 0.45 | 1 | 0.25 | 0.7 | 0.55 | 1 | 0.25 |
| P24 | M | 63.7 | 385.1 | 14 | S | 3 | 0.76 | 0.5 | 1 | 0.31 | 0.24 | 0.5 | 0.48 | 0.31 |
| P25 | M | 55.7 | 46.6 | 12 | E | 5 | 0.71 | 0.93 | 0.8 | 0.82 | 0.29 | 0.07 | 0.77 | 0.71 |
| P26 | F | 67.8 | 8.3 | 15 | S | 21 | 0.77 | 0.39 | 1 | 0.48 | 0.23 | 0.61 | 0.92 | 0.31 |
| P27 | F | 50.6 | 31.7 | 17 | S | 16 | 0.47 | 0.99 | 1 | 0.6 | 0.53 | 0.01 | 0.77 | 0.28 |
| P28 | F | 44.5 | 34.6 | 17 | E | 0 | 0.93 | 0.94 | 1 | 0.22 | 0.07 | 0.06 | 0.82 | 0.17 |
| P29 | M | 53.5 | 6.8 | 12 | S | 9 | 0.05 | 0.5 | 1 | 1 | 0.95 | 0.5 | 1 | 1 |
| P30 | M | 58.8 | 8.9 | 7 | S | 17 | 0.96 | 1 | 1 | 0.57 | 0.04 | 0 | 0.82 | 0.34 |
| P31 | F | 38.4 | 7 | 16 | S | 8 | 0.26 | 0.1 | 1 | 0.51 | 0.74 | 0.9 | 1 | 0.51 |
| P32 | M | 59.9 | 30 | 12 | S | 5 | 0.5 | 0.23 | 1 | 0.54 | 0.5 | 0.77 | 1 | 0.65 |
| P33 | F | 49.4 | 17.1 | 13 | S | 3 | 0.52 | 0.07 | 0.77 | 0.31 | 0.48 | 0.93 | 1 | 0.34 |
| P34 | M | 33 | 9.9 | 10 | S | 24 | 0.82 | 0.89 | 1 | 0.71 | 0.18 | 0.11 | 0.37 | 0.25 |
| P35 | F | 57 | 69.9 | 14 | S | 1 | 0.14 | 0.32 | 0.6 | 0.2 | 0.86 | 0.68 | 1 | 0.8 |
| P36 | M | 62.5 | 9.5 | 13 | S | 35 | 0.87 | 0.98 | 1 | 0.71 | 0.13 | 0.02 | 0.62 | 0.62 |
| P37 | M | 59.3 | 8.8 | 12 | S | 0 | 0.35 | 0.25 | 1 | 0.48 | 0.65 | 0.75 | 1 | 0.48 |
| P38 | M | 18.7 | 13.9 | 13 | S | 3 | 0.36 | 0.07 | 1 | 0.38 | 0.64 | 0.93 | 1 | 0.55 |
| P39 | F | 73.2 | 64.1 | 12 | S | 5 | 0.74 | 0.6 | 1 | 0.74 | 0.26 | 0.4 | 1 | 0.8 |
| P40 | M | 57.1 | 7.2 | 15 | S | 27 | 0.65 | 0.79 | 1 | 0.65 | 0.35 | 0.21 | 0.6 | 0.48 |
| P41 | M | 76.9 | 12.5 | 10 | S | 55 | 0.35 | 0.75 | 1 | 0.54 | 0.65 | 0.25 | 0.48 | 0.2 |
| P42 | F | 37.4 | 20.6 | 17 | S | 8 | 0.45 | 0.28 | 1 | 0.94 | 0.55 | 0.72 | 0.94 | 0.88 |
| P43 | M | 64.5 | 6.1 | 12 | S | 32 | 0.71 | 1 | 1 | 0.48 | 0.29 | 0 | 0.65 | 0.4 |
| P44 | M | 76.8 | 10.8 | 18 | E | 18 | 0.94 | 0.95 | 1 | 0.64 | 0.06 | 0.95 | 0.94 | 0.55 |
| P45 | M | 31.7 | 36.5 | 5 | S | 21 | 0.76 | 1 | 1 | 0.4 | 0.24 | 0 | 0.34 | 0.25 |
| P46 | M | 49.2 | 11.2 | 18 | S | 13 | 0.5 | 0.62 | 1 | 0.31 | 0.5 | 0.38 | 1 | 0.31 |
| P47 | M | 47.7 | 6.4 | 22 | S | 6 | 0.17 | 0.73 | 1 | 0.8 | 0.83 | 0.27 | 1 | 0.77 |
| P48 | M | 63 | 20.2 | 19 | E | 21 | 1 | 1 | 1 | 0.97 | 0 | 0 | 0.6 | 0.37 |

Note. Values are provided in Means (M) and Standard Deviations (SD). *MPO* = Months post-onset; *Edu* = Education in years; *L1* = first acquired language; *L2* = second acquired language; *AoA* = age of second language acquisition; *S* = Spanish, *E* = English; *Language Use* = proportion of time spent using each language in a typical week; *Language Ability Rating* = self-reported percentage score of language skills in each language where closer to 1 means stronger skills; *Pre* = pre-stroke; *Post* = post-stroke. P01 and P22 did not complete all sections of the Language Use Questionnaire and these data are marked *NA*.

| Supplementary Table S2. *Individual clinical assessment scores and treatment language information* | | | | | | | | | | | | |
| --- | --- | --- | --- | --- | --- | --- | --- | --- | --- | --- | --- | --- |
| ID |  |  |  | | L1 | | | | L2 | | | |
|  |  | PAPT (%) | | | AQ | | BNT (%) | | AQ | | BNT (%) | |
|  | Tx Lang | Pre | | Post | Pre | Post | Pre | Post | Pre | Post | Pre | Post |
| P01 | S | 0.42 | | NA | 55.7 | 56.8 | 0.13 | 0.1 | 29.6 | 31.9 | 0.01 | 0.01 |
| P02 | S | 0.87 | | 0.9 | 74.1 | 74 | 0.36 | 0.46 | 68.5 | 78 | 0.38 | 0.43 |
| P03 | S | 0.9 | | 0.98 | 84.5 | 84.5 | 0.46 | 0.48 | 89.8 | 93.5 | 0.78 | 0.75 |
| P04 | E | 0.81 | | NA | 37.3 | 56.8 | 0.28 | 0.21 | 27.3 | 28.7 | 0.01 | 0.01 |
| P05 | S | 0.88 | | 0.88 | 77.5 | 79.5 | 0.35 | 0.4 | 67.6 | 76.6 | 0.41 | 0.5 |
| P06 | S | 0.92 | | 0.94 | 82 | 83.8 | 0.51 | 0.5 | 67.6 | 78.8 | 0.4 | 0.28 |
| P07 | S | 0.94 | | 0.98 | 79.1 | 79.9 | 0.63 | 0.6 | 54.4 | 56.9 | 0.13 | 0.18 |
| P08 | S | 0.88 | | 0.92 | 51.3 | 53.9 | 0.06 | 0.26 | 47.5 | 53.8 | 0.21 | 0.2 |
| P09 | S | 0.96 | | 0.94 | 67.4 | 71.7 | 0.51 | 0.51 | 64.7 | 78.6 | 0.5 | 0.55 |
| P10 | E | 0.92 | | 0.92 | 76.8 | 79.4 | 0.4 | 0.38 | 78.9 | 78.8 | 0.45 | 0.38 |
| P11 | S | 0.92 | | 0.88 | 57.3 | 71 | 0.36 | 0.43 | 39.8 | 41.7 | 0.08 | 0.16 |
| P12 | S | 0.81 | | 0.82 | 72.3 | 74.1 | 0.23 | 0.21 | 66.4 | 69.8 | 0.15 | 0.25 |
| P13 | S | 0.98 | | 0.96 | 68.8 | 79.4 | 0.4 | 0.43 | 90 | 93.2 | 0.9 | 0.91 |
| P14 | E | 0.92 | | 0.92 | 46.5 | 43.3 | 0.18 | 0.25 | 35.9 | 40 | 0.1 | 0.1 |
| P15 | E | 0.94 | | 0.98 | 96.5 | 99.4 | 0.9 | 0.91 | 60.8 | 53 | 0.13 | 0.15 |
| P16 | E | 0.69 | | 0.63 | 11.4 | 5.2 | 0 | 0 | 9.5 | 7.8 | 0 | 0 |
| P17 | S | 0.87 | | NA | 82.4 | 66.3 | 0.53 | 0.55 | 71.2 | 59.5 | 0.38 | 0.4 |
| P18 | E | 0.92 | | 0.92 | 91 | 94.6 | 0.8 | 0.8 | 81.2 | 89.8 | 0.31 | 0.43 |
| P19 | E | 0.88 | | 0.86 | 39.5 | 34.9 | 0.05 | 0.06 | 21 | 32.8 | 0.06 | 0.06 |
| P20 | E | 0.9 | | 0.92 | 94.6 | 93.5 | 0.68 | 0.76 | 57.8 | 73.8 | 0.16 | 0.11 |
| P21 | S | 0.96 | | 0.98 | 78.6 | 81.7 | 0.38 | 0.5 | 85.2 | 89.9 | 0.83 | 0.75 |
| P22 | S | 0.73 | | NA | 7.1 | 8.6 | 0 | NA | 10.1 | 8.6 | 0 | NA |
| P23 | E | 0.88 | | 0.82 | 53.3 | 65.6 | 0.31 | 0.26 | 34.4 | NA | 0.06 | 0.01 |
| P24 | E | 0.73 | | 0.32 | 47.7 | 40.5 | 0.36 | 0.3 | 15.4 | 15.6 | 0.01 | 0 |
| P25 | E | 0.94 | | 0.94 | 97.2 | 96.2 | 0.85 | 0.85 | 92.6 | 91.3 | 0.55 | 0.61 |
| P26 | E | 0.46 | | NA | 23.9 | 8.8 | 0 | 0 | 21.7 | 29.1 | 0 | 0 |
| P27 | S | 0.98 | | 0.98 | 92.6 | NA | 0.53 | 0.61 | 69.8 | NA | 0.33 | 0.4 |
| P28 | E | 0.92 | | 0.94 | 85 | 91 | 0.68 | 0.63 | 36.5 | 43.8 | 0.08 | 0.1 |
| P29 | E | 0.81 | | 0.84 | 40.1 | 60.3 | 0.23 | 0.25 | 41.4 | 37.1 | 0.05 | 0.08 |
| P30 | E | 0.58 | | 0.84 | 7.8 | 9.6 | 0 | 0 | 14.7 | 14.1 | 0 | 0 |
| P31 | E | 0.88 | | 0.88 | 82.6 | 91.4 | 0.61 | 0.76 | 68.3 | 74.8 | 0.43 | 0.45 |
| P32 | S | 0.88 | | NA | 21.7 | NA | 0 | NA | 50.9 | NA | 0.25 | 0.36 |
| P33 | S | 0.85 | | 0.8 | 7.6 | 9.5 | 0 | 0 | 10.9 | 11.3 | 0 | 0.01 |
| P34 | S | 0.83 | | 0.9 | 76.1 | 87.1 | 0.51 | 0.48 | 20.5 | 29.9 | 0.01 | 0.01 |
| P35 | E | 0.88 | | 0.88 | 89.6 | 92.1 | 0.8 | 0.81 | 60.5 | 65.8 | 0.15 | 0.21 |
| P36 | E | 0.94 | | 1 | 61.7 | 64.1 | 0.36 | 0.38 | 85.4 | 85.1 | 0.7 | 0.75 |
| P37 | S | 0.65 | | 0.34 | 15.8 | 11 | 0 | 0 | 10.6 | 9.9 | 0 | 0 |
| P38 | E | 0.92 | | 0.92 | 72.6 | 79.8 | 0.35 | 0.41 | 27 | 27.7 | 0.01 | 0.01 |
| P39 | E | 0.94 | | 0.98 | 93.8 | 92.9 | 0.78 | 0.76 | 92.4 | 89.5 | 0.6 | 0.51 |
| P40 | S | 0.62 | | 0.59 | 19.6 | 26.1 | 0 | 0.03 | 14.7 | 18.3 | 0 | 0 |
| P41 | S | 0.75 | | 0.8 | 90.8 | 93.1 | 0.56 | 0.55 | 46.3 | 65.8 | 0.2 | 0.21 |
| P42 | S | 0.96 | | 0.92 | 90.2 | 91.8 | 0.6 | 0.68 | 84 | 79.6 | 0.46 | 0.46 |
| P43 | S | 0.42 | | NA | 15.7 | 28.3 | 0.03 | 0.1 | 9.9 | 8.7 | 0 | 0 |
| P44 | S | 0.85 | | 0.73 | 39.7 | 49 | 0 | 0.01 | 55.8 | 57.9 | 0.01 | 0.05 |
| P45 | S | 0.62 | | 0.57 | 22.6 | 26.5 | 0.03 | 0.1 | 18.3 | 13.9 | 0 | 0 |
| P46 | S | 0.83 | | 0.84 | 14 | 22.4 | 0.01 | 0.03 | 13 | 18.4 | 0.03 | 0.03 |
| P47 | S | 0.96 | | 0.9 | 94.4 | 98.4 | 0.85 | 0.9 | 93.9 | 97.4 | 0.71 | 0.8 |
| P48 | S | 0.98 | | 0.96 | 64.3 | 79.3 | 0.13 | 0.3 | 97.8 | 97.8 | 0.91 | 0.95 |
| Note. Values are provided in Means (M) and (SD). *PAPT =* Pyramids and Palm Trees Percentage Score (/52); *AQ =* Aphasia Quotient, a measure of aphasia severity from the Western Aphasia Battery-Revised (/100); *BNT =* Boston Naming Test Percentage Score (/60); *Tx Lang* = Treatment Language; *S* = Spanish; *E* = English. Some participants exited the study before completing all post-treatment assessments. *NA* = Not Assessed | | | | | | | | | | | | |

| Supplementary Table S3. *Description of treatment steps* | |
| --- | --- |
| Treatment Step | Description |
| 1. Naming | The participant was shown a picture and asked to name it |
| 2. Feature Classification | The participant was shown a list of 15 semantic features that may or may not apply to the picture |
| 2A. Feature Selection | In selection, participants were asked to identify all features /15 that DID NOT apply to the pictured item |
| 2B. Feature Assignment | In assignment, participants were asked to sort the remaining affirmative features into boxes that explained their relationship to the pictured item (function, characteristics, physical attributes, location, and superordinate category) |
| 3. Association | The participant was shown a picture of the item and asked to generate another item or experience that the picture called to mind; the participant was directed to explain the association to the clinician |
| 4. Yes/No questions | The participant was again shown 15 features and asked to distinguish between those that applied to the picture (yes) and those that did not (no) |
| 5. Naming | The participant was again shown the picture and asked to name it |
| 6. Sentence Production | The participant was asked to use the name of the picture in a sentence to illustrate its meaning |
| Note. Participants were shown a maximum of 15 items each treatment session. All 6 steps would be completed for each item before moving on to the next item. The picture for each item was visible during all treatment steps and was always the same session to session. | |

| Supplementary Table S4. *Error type intercept and slope predictions* | | | | | |
| --- | --- | --- | --- | --- | --- |
| Term | Parameter | Coefficient  (SE) | IRR | *z* | *p* adj.  (*q*) |
| Model 1: Trained Words in the Treated Language | | | | | |
| No Response | Intercept | 1.39 (0.10) | 4.01 | 12.81 |  |
|  | Slope | -0.08 (0.01) | 0.91 | -6.61 | **<.001** |
| Neologism | Intercept | 0.17 (0.11) | 1.18 | 1.46 |  |
|  | Slope | 0.009 (0.01) | 1.00 | 0.72 | .517 |
| Perseveration | Intercept | -0.58 (0.14) | 0.55 | -4.13 |  |
|  | Slope | -0.03 (0.01) | 0.96 | -2.27 | **.033** |
| Unrelated | Intercept | -0.49 (0.13) | 0.61 | -3.65 |  |
|  | Slope | -0.007 (0.01) | 0.99 | -0.47 | .632 |
| Circumlocution | Intercept | 0.52 (0.12) | 1.68 | 4.22 |  |
|  | Slope | -0.15 (0.01) | 0.85 | -9.33 | **<.001** |
| Semantic | Intercept | 1.14 (0.11) | 3.14 | 10.10 |  |
|  | Slope | -0.13 (0.01) | 0.87 | -9.63 | **<.001** |
| Mixed | Intercept | 0.07 (0.12) | 1.08 | 0.61 |  |
|  | Slope | -0.10 (0.01) | 0.90 | -6.13 | **<.001** |
| Phonological | Intercept | -1.37 (0.16) | 0.25 | -8.25 |  |
|  | Slope | 0.01 (0.01) | 1.01 | 0.99 | .398 |
| Correct (Non-Target) | Intercept | -0.86 (0.17) | 0.42 | -5.01 |  |
|  | Slope | -0.16 (0.02) | 0.85 | -6.12 | **<.001** |
| Correct (Target) | Intercept | 1.10 (0.10) | 3.01 | 10.37 |  |
|  | Slope | 0.10 (0.01) | 1.11 | 8.85 | **<.001** |
| Model 2: Semantically Related Words in the Treated Language | | | | | |
| No Response | Intercept | 1.36 (0.09) | 3.91 | 14.58 |  |
|  | Slope | -0.01 (0.01) | 0.98 | -1.27 | .290 |
| Neologism | Intercept | 0.16 (0.10) | 1.17 | 1.54 |  |
|  | Slope | 0.01 (0.01) | 1.01 | 1.55 | .290 |
| Perseveration | Intercept | -0.50 (0.12) | 0.60 | -3.95 |  |
|  | Slope | -0.03 (0.01) | 0.96 | -2.40 | .054 |
| Unrelated | Intercept | -0.41 (0.12) | 0.65 | -3.37 |  |
|  | Slope | -0.01 (0.01) | 0.98 | -1.27 | .290 |
| Circumlocution | Intercept | 0.46 (0.10) | 1.59 | 4.47 |  |
|  | Slope | -0.03 (0.01) | 0.96 | -2.57 | .050 |
| Semantic | Intercept | 1.24 (0.09) | 3.46 | 13.19 |  |
|  | Slope | -0.001 (0.01) | 0.99 | -0.15 | .880 |
| Mixed | Intercept | 0.02 (0.11) | 1.02 | 0.18 |  |
|  | Slope | -0.01 (0.01) | 0.98 | -1.30 | .290 |
| Phonological | Intercept | -1.48 (0.16) | 0.22 | -8.74 |  |
|  | Slope | -0.003 (0.01) | 0.99 | -0.19 | .880 |
| Correct (Non-Target) | Intercept | -1.10 (0.14) | 0.33 | -7.59 |  |
|  | Slope | 0.01 (0.01) | 1.01 | 0.99 | .400 |
| Correct (Target) | Intercept | 0.74 (0.09) | 2.10 | 7.65 |  |
|  | Slope | 0.03 (0.01) | 1.03 | 3.49 | **<.01** |
| Model 3: Control Words in the Treated Language | | | | | |
| No Response | Intercept | 1.34 (0.09) | 3.85 | 13.7 |  |
|  | Slope | -0.01 (0.01) | 0.98 | -1.74 | .271 |
| Neologism | Intercept | 0.34 (0.10) | 1.41 | 3.19 |  |
|  | Slope | 0.003 (0.01) | 1.00 | 0.29 | .839 |
| Perseveration | Intercept | -0.42 (0.12) | 0.65 | -3.28 |  |
|  | Slope | -0.04 (0.01) | 0.95 | -2.68 | **.036** |
| Unrelated | Intercept | -0.39 (0.12) | 0.67 | -3.22 |  |
|  | Slope | 0.002 (0.01) | 1.00 | 0.20 | .839 |
| Circumlocution | Intercept | 0.31 (0.10) | 1.37 | 2.88 |  |
|  | Slope | -0.01 (0.01) | 0.98 | -1.35 | .441 |
| Semantic | Intercept | 1.16 (0.09) | 3.19 | 11.68 |  |
|  | Slope | -0.009 (0.01) | 0.99 | -0.81 | .590 |
| Mixed | Intercept | -0.15 (0.11) | 0.86 | -1.27 |  |
|  | Slope | -0.005 (0.01) | 0.99 | -0.42 | .838 |
| Phonological | Intercept | -1.58 (0.18) | 0.20 | -8.68 |  |
|  | Slope | -0.02 (0.02) | 0.97 | -0.99 | .532 |
| Correct (Non-Target) | Intercept | -1.15 (0.15) | 0.31 | -7.70 |  |
|  | Slope | 0.01 (0.01) | 1.01 | 1.16 | .492 |
| Correct (Target) | Intercept | 0.92 (0.10) | 2.52 | 9.25 |  |
|  | Slope | 0.03 (0.01) | 1.03 | 3.44 | **<.01** |
| Model 4: Translations of Trained Words in the Untreated Language | | | | | |
| No Response | Intercept | 1.71 (0.11) | 5.53 | 15.07 |  |
|  | Slope | -0.01 (0.01) | 0.98 | -1.13 | .319 |
| Neologism | Intercept | 0.26 (0.12) | 1.30 | 2.13 |  |
|  | Slope | 0.01 (0.01) | 1.01 | 0.74 | .456 |
| Perseveration | Intercept | -0.65 (0.14) | 0.51 | -4.37 |  |
|  | Slope | -0.03 (0.01) | 0.96 | -2.12 | .055 |
| Unrelated | Intercept | -0.48 (0.14) | 0.61 | -3.42 |  |
|  | Slope | -0.01 (0.01) | 0.98 | -0.74 | .456 |
| Circumlocution | Intercept | 0.32 (0.12) | 1.38 | 2.54 |  |
|  | Slope | -0.07 (0.01) | 0.93 | -4.65 | **<.001** |
| Semantic | Intercept | 0.91 (0.12) | 2.48 | 7.52 |  |
|  | Slope | -0.08 (0.01) | 0.92 | -5.72 | **<.001** |
| Mixed | Intercept | -0.21 (0.13) | 0.80 | -1.54 |  |
|  | Slope | -0.04 (0.01) | 0.95 | -2.55 | **.021** |
| Phonological | Intercept | -1.95 (0.20) | 0.14 | -9.58 |  |
|  | Slope | 0.03 (0.02) | 1.03 | 1.75 | .112 |
| Correct (Non-Target) | Intercept | 0.06 (0.12) | 1.07 | 0.56 |  |
|  | Slope | 0.07 (0.01) | 1.08 | 5.64 | **<.001** |
| Correct (Target) | Intercept | 0.27 (0.12) | 1.31 | 2.24 |  |
|  | Slope | 0.07 (0.01) | 1.08 | 5.82 | **<.001** |
| Model 5: Translations of Semantically Related Words in the Untreated Language | | | | | |
| No Response | Intercept | 1.68 (0.10) | 5.40 | 16.33 |  |
|  | Slope | -0.006 (0.01) | 0.99 | -0.58 | .701 |
| Neologism | Intercept | 0.28 (0.11) | 1.32 | 2.44 |  |
|  | Slope | 0.01 (0.01) | 1.01 | 0.96 | .475 |
| Perseveration | Intercept | -0.58 (0.13) | 0.55 | -4.17 |  |
|  | Slope | -0.03 (0.01) | 0.96 | -2.18 | .096 |
| Unrelated | Intercept | -0.47 (0.13) | 0.62 | -3.53 |  |
|  | Slope | -0.01 (0.01) | 0.98 | -0.96 | .475 |
| Circumlocution | Intercept | 0.25 (0.11) | 1.28 | 2.12 |  |
|  | Slope | -0.03 (0.01) | 0.96 | -2.50 | .061 |
| Semantic | Intercept | 1.04 (0.10) | 2.83 | 9.73 |  |
|  | Slope | -0.004 (0.01) | 0.99 | -0.35 | .725 |
| Mixed | Intercept | -0.16 (0.12) | 0.85 | -1.30 |  |
|  | Slope | -0.006 (0.01) | 0.99 | -0.42 | .725 |
| Phonological | Intercept | -1.83 (0.19) | 0.15 | -9.50 |  |
|  | Slope | 0.02 (0.02) | 1.02 | 1.13 | .475 |
| Correct (Non-Target) | Intercept | -0.58 (0.13) | 0.55 | -4.39 |  |
|  | Slope | 0.02 (0.01) | 1.02 | 1.56 | .294 |
| Correct (Target) | Intercept | 0.37 (0.11) | 1.45 | 3.35 |  |
|  | Slope | 0.03 (0.01) | 1.03 | 3.06 | **<.05** |
| Model 6: Translations of Control Words in the Untreated Language | | | | | |
| No Response | Intercept | 1.67 (0.10) | 5.36 | 15.65 |  |
|  | Slope | -0.01 (0.01) | 0.98 | -1.01 | .518 |
| Neologism | Intercept | 0.29 (0.11) | 1.34 | 2.48 |  |
|  | Slope | 0.01 (0.01) | 1.01 | 0.87 | .547 |
| Perseveration | Intercept | -0.60 (0.14) | 0.54 | -4.24 |  |
|  | Slope | -0.02 (0.01) | 0.97 | -1.65 | .292 |
| Unrelated | Intercept | -0.36 (0.13) | 0.69 | -2.75 |  |
|  | Slope | -0.007 (0.01) | 0.99 | -0.47 | .713 |
| Circumlocution | Intercept | 0.09 (0.12) | 1.10 | 0.77 |  |
|  | Slope | -0.02 (0.01) | 0.97 | -1.55 | .297 |
| Semantic | Intercept | 1.01 (0.11) | 2.76 | 9.14 |  |
|  | Slope | -0.005 (0.01) | 0.99 | -0.46 | .713 |
| Mixed | Intercept | -0.22 (0.12) | 0.80 | -1.71 |  |
|  | Slope | 0.00 (0.01) | 1.00 | 0.01 | .990 |
| Phonological | Intercept | -1.90 (0.20) | 0.14 | -9.52 |  |
|  | Slope | 0.02 (0.02) | 1.02 | 1.13 | .517 |
| Correct (Non-Target) | Intercept | -0.27 (0.12) | 0.75 | -2.16 |  |
|  | Slope | 0.02 (0.01) | 1.02 | 2.03 | .208 |
| Correct (Target) | Intercept | 0.39 (0.11) | 1.47 | 3.37 |  |
|  | Slope | 0.03 (0.01) | 1.03 | 2.79 | .051 |
| Note. Intercept and Slope estimates were obtained from contrast matrices for each individual word set x language combination. The values for each error type were computed independently of each other to provide straightforward interpretability of the effects on error counts. *Coefficient* = Log(Predictor Variable); *SE* = Standard Error; *IRR* = Incidence Rate Ratio: The log coefficients in count regression models may be exponentiated to retrieve a ratio of how often an event occurs (e.g., errors in a given session); *z* = test-statistic associated with generalized linear modeling; *q* = false-detection rate, adjusted p-value with significance threshold at .05. | | | | | |
